# A metabolic profile in *Ruditapes philippinarum* associated with growth-promoting effects of alginate hydrolysates

**DOI:** 10.1038/srep29923

**Published:** 2016-07-20

**Authors:** Yasuhiro Yamasaki, Shigeru Taga, Masanobu Kishioka, Shuichi Kawano

**Affiliations:** 1 Laboratory of Environmental Biology, Department of Applied Aquabiology, National Fisheries University, Yamaguchi, Japan; 2 Yamaguchi Prefectural Fisheries Research Center, Yamaguchi, Japan; 3 Graduate School of Informatics and Engineering, The University of Electro-Communications, Tokyo, Japan

## Abstract

The aim of this study is to demonstrate the growth-promoting effect of alginate hydrolysates (AHs) on the Manila clam *Ruditapes philippinarum*, and to verify the physiological change occurring within a living *R. philippinarum* stimulated by AHs. We show that growth of clams was dramatically promoted by supplementing a diet of the diatom *Chaetoceros neogracile* with AHs at 4 mg/mL. Furthermore, metabolomics indicates that each state of starvation, food satiation, and sexual maturation have a characteristic pattern. In the groups given AHs in addition to *C. neogracile* in particular, excess carbohydrate was actively utilized for the development of reproductive tissue. In contrast, it appeared that clams in the groups given *C. neogracile* only were actively growing, utilizing their adequate carbohydrate resources. Meanwhile, the unfed groups have slowed growth because of the lack of an energy source. Hence, supplementation of AHs in addition to the algal diet may be an inexpensive way to shorten the rearing period of *R. philippinarum*. Moreover, metabolomics can evaluate the growth condition of *R. philippinarum* in a comprehensive way, and this approach is crucially important for not only the development of a mass culture method but also for the conservation of the clam resource in the field.

Suspension-feeding bivalves are considered “keystone” species in freshwater and coastal marine environments^1^. These filter-feeders can exert “top-down” grazer control on phytoplankton and reduce turbidity^2^. This can increase the amount of light reaching the sediment and enable the growth of benthic plants such as seagrasses and benthic microalgae^2^. Furthermore, suspension-feeding bivalves assume “bottom-up” control through biodeposition and promotion of nutrient removal, and stabilization of phytoplankton growth dynamics through the moderation of ammonia cycling in the water column^1^; thus these species play a uniquely important role in the ecosystem.

The Manila clam *Ruditapes philippinarum* (Adams and Reeve, 1850) is well known as an ecologically important bivalve as a filter feeder. Originally, wild populations of *R. philippinarum* were found in the Philippines, the South and East China seas, Yellow Sea, Sea of Japan, Sea of Okhotsk, and around the Southern Kuril Islands; it is known as a subtropical to low boreal species^3^. In the 1930s, *R. philippinarum* was accidentally introduced to the Pacific coast of North America along with seed of the Pacific cupped oyster *Crassostrea gigas*, whereas this species was deliberately introduced to European waters from the 1970s onward because it has a high commercial value^3^. Today, *R. philippinarum* is not only a commercially important bivalve but also one of most ecologically important bivalves in the world, and production of this species reached approximately 4 million tonnes in 2013^3^.

However, the annual catch of this species in coastal waters of Japan, which once led the world, continues to decrease drastically. Several factors have been suggested as causes of the dramatic decrease^4^,^5^,^6^,^7^,^8^ in addition to overfishing, although the precise cause is as yet unknown. On the other hand, there have been a wide variety of studies conducted to achieve the conservation of the clam resource and the development of clam culture^9^,^10^,^11^,^12^,^13^. With the present-day, relatively rapid global environmental change, however, there is the potential for the annual catch of *R. philippinarum* in other countries to decrease dramatically as it has in Japan. Hence, the International Symposium on the Manila (Asari) Clam convened in 2008, 2012, and 2015, and the current status of Manila clam production among countries was discussed for a better understanding of the issues and a resolution on clam production. Thus, the development of a growth-promoting factor, a mass-culture method for production of the clam, and clarification of its growth mechanisms would have important implications for clam culture, and would contribute to the conservation of the clam resource in the field and a stable market supply.

Recently, Uchida *et al*. reported that the growth rate of soft tissue in *R. philippinarum* was significantly promoted by supplementing a diet of the diatom *Chaetoceros calcitrans* with glucose^14^. Thus, certain types of sugars are potentially a good supplement for *R. philippinarum* growth. Furthermore, Taga *et al*. reported the beneficial effects of the raphidophyte *Heterosigma akashiwo*, known as a harmful algal species, on the diet of juvenile *R. philippinarum*, and suggested the possibility that certain kinds of sugars, specifically the acidic sugars in phytoplankton, are one of the important factors determining the growth of juvenile clams^15^.

Alginate is a natural acidic linear polysaccharide that is composed of α-l-guluronate and β-d-mannuronate (uronic acids) residues, and is also known as a type of dietary fiber. It is well known that the outer layer of the kelp *Laminaria japonica* and the brown seaweed *Undaria pinnatifida* has high-viscosity because alginate fills a gap between adjoining cell walls of these brown algae. In addition, several studies have reported that the compositional ratio of α-l-guluronate and β-d-mannuronate or the degree of polymerization affect the physical properties and multiple biological activities of the alginate^16^,^17^. Therefore, we focused on one of the acidic polysaccharides, alginate, and its derivatives, which are currently used in a wide range of commercial enterprises, including the food, medical, cosmetic and textile-processing industries. Additionally, the dietary administration of alginate stimulates the immune abilities of white shrimp and juvenile carp^18^,^19^. Preliminary observations by Yamasaki *et al*. suggested that growth of clams was significantly promoted by supplementing a diet of *Chaetoceros neogracile* (diatom) with alginate-hydrolysates (AHs) of at least 1 mg/L; the most effective concentration of AHs was 2 to 4 mg/L^20^. What is interesting about this finding is the underlying mechanism of growth promotion in *R. philippinarum* because alginate, a known dietary fiber, is not likely to be the energy source for the clam growth. In fact, the growth promoting effect did not find in the groups given AHs only^20^.

In this study, we demonstrated the growth-promoting effect of AHs on *R. philippinarum* in order to develop a method of clam culture. In addition, to understand the mechanism behind this effect, we used metabolomics, which has received attention in recent years for the following four reasons; 1) the number of target substances is significantly lower than other “-omics” such as transcriptomics and proteomics; 2) targets are low-molecular-weight compounds about which much is known from the published literature on their physiology and pathology; 3) it can make a direct observation of phenomena occurring within a living organism (i.e., changes in a metabolome mean changes in enzymatic activity); and 4) results are not usually species specific (i.e. results from one species are usually applicable to other species.). Therefore, we tried an exhaustive analysis for a metabolic signature in *R. philippinarum* stimulated by AHs by utilizing a capillary electrophoresis time-of-flight mass spectrometry (CE-TOFMS) with the goal of characterizing the relationship between the metabolome and growth of *R. philippinarum*.

## Results

### Growth-promoting effect of AHs on clam growth

**Figure 1:**
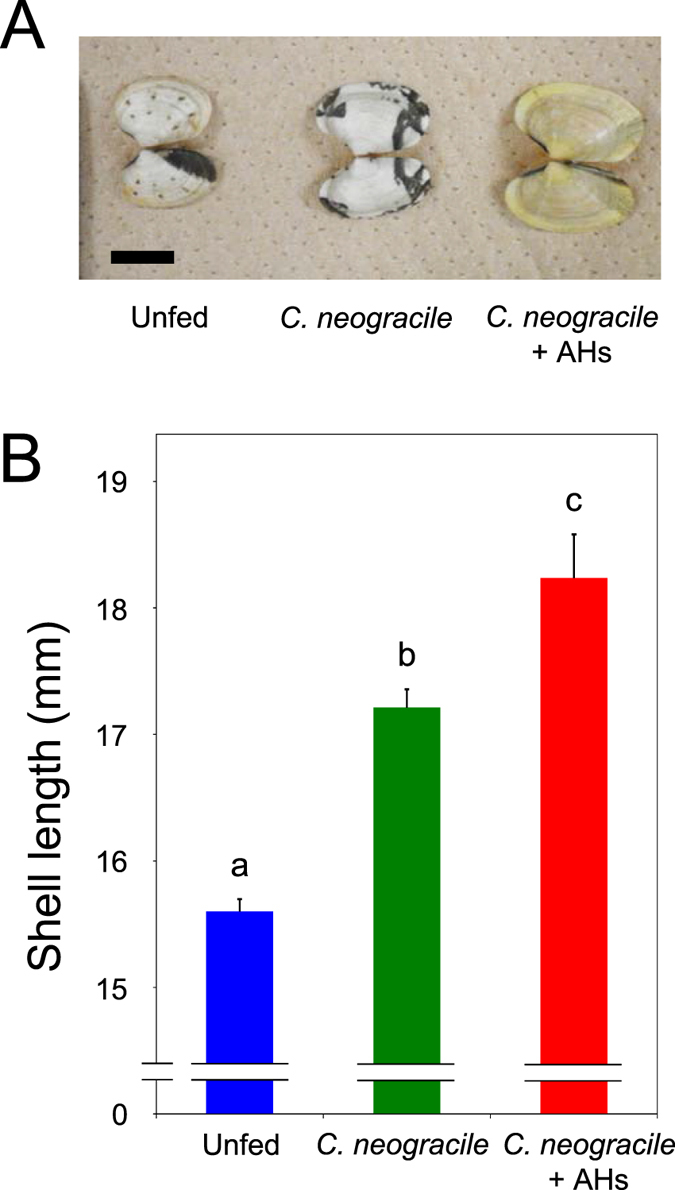
Effect of AHs on shell length of the Manila clam *R. philippinarum*. (**A**) Representative largest clams from each feeding group. Scale bar = 10 mm. (**B**) Average shell length of control and experimental feeding groups. Rearing tests were conducted as described in the text. Data are means ± SD of individuals in each test group (*n* = 3). Bars with different lower-case letters are significantly different at *P* < 0.05 (one-way analysis of variance (ANOVA) and then tested by using Tukey’s post hoc test).

**Figure 2:**
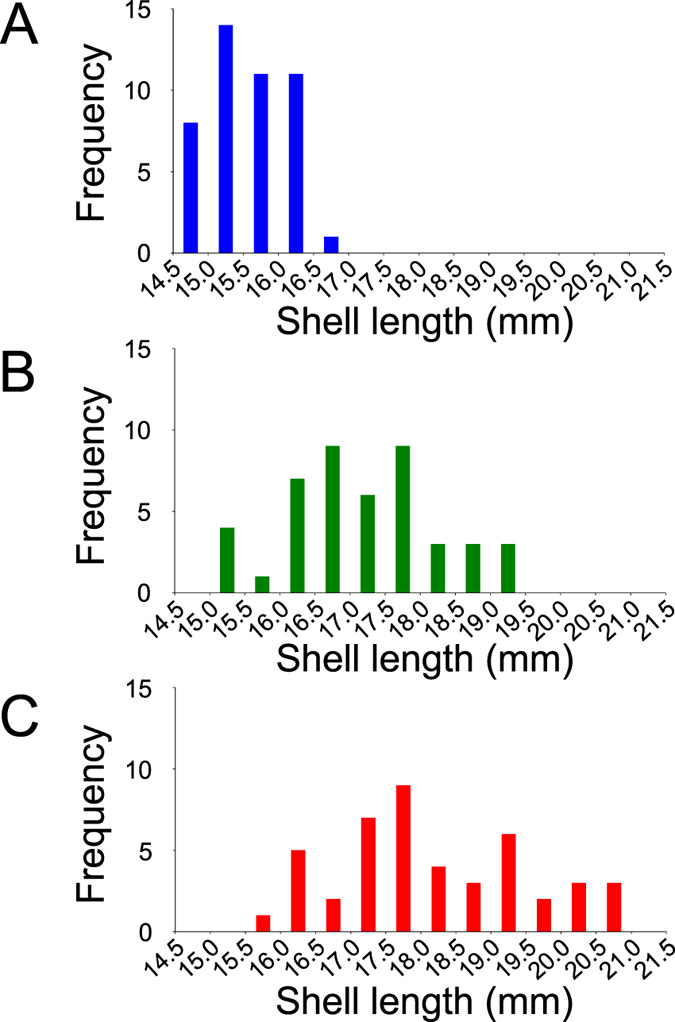
Length–frequency histograms of the Manila clam *R. philippinarum*. (**A**) Length–frequency histograms of the no-diet groups. (**B**) Length–frequency histograms of the groups given only *C. neogracile*. (**C**) Length–frequency histograms of the groups given AHs (4 mg/mL) in addition to *C. neogracile*. Rearing tests were conducted as described in the text.

Growth of clams was dramatically promoted by supplementing a diet of *C. neogracile* with AHs at 4 mg/mL (Fig. 1). The growth-promoting effect of AHs on the clams is clearly evident in a visual comparison of maximum shell length in each test group (Fig. 1A). In addition, the average shell length in the groups given AHs in addition to *C. neogracile* was significantly greater than those in the other test groups (Fig. 1B, *P* < 0.05). Although the shell lengths in unfed clams at the end of the rearing experiment were almost the same as at the beginning, groups given any diet showed an increase in shell-length (Fig. 1B). In the groups given AHs in addition to *C. neogracile* in particular, remarkable growth was observed in some clams (Fig. 1B). Furthermore, length–frequency histograms of each group indicated the growth-promoting effect of AHs on the clams (Fig. 2). The mode in unfed clams was more than 15.0 mm but less than 15.5 mm (Fig. 2A) and in the groups given AHs in addition to *C. neogracile* was more than 17.5 mm but less than 18.0 mm (Fig. 2C). In addition, the modes in the groups given *C. neogracile* were more than 16.5 mm but less than 17.0 mm and more than 17.5 mm but less than 18.0 mm (Fig. 2B).

In addition to shell length, there were significant differences in shell height, shell width, weight, roundness index, and maturity index between test groups (Table 1, *P* < 0.05). There were no dead clams observed in any test group during the rearing experiment.

### Heat map and PCA representation of metabolome data from clams under different rearing conditions

**Figure 3:**
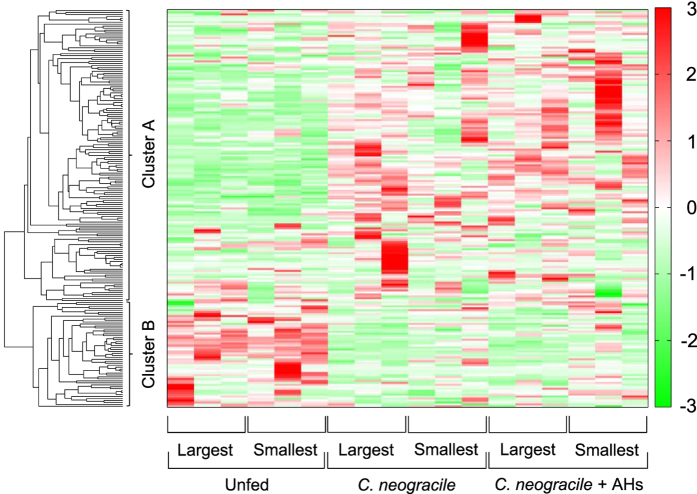
Heat map of metabolites in the no-diet groups, the groups given only *C. neogracile*, and the groups given *C. neogracile* plus AHs (4 mg/mL). The color scale from green to red indicates low to high correlation, respectively. Hierarchical cluster analysis was conducted as described in the text.

Two hundred and three metabolites (116 cation, 87 anion) were detected by using CE-TOFMS in clams reared under the various conditions (see Supplementary Appendix S1), and 87 of the 203 metabolites (47 cation, 40 anion) were quantified (see Supplementary Appendix S2). The HCA and heat map revealed a sharp contrast between the no-diet groups and the groups fed *C. neogracile* (Fig. 3). In particular, metabolites in clusters A and B clearly showed the difference between these groups (Fig. 3). Incidentally, cluster B had a higher tendency to include metabolites involved in amino acid metabolism, whereas cluster A showed little tendency to include particular metabolites. The heat map did not show any clear difference between the groups given *C. neogracile* only and the groups given AHs in addition to *C. neogracile* (Fig. 3).

**Figure 4:**
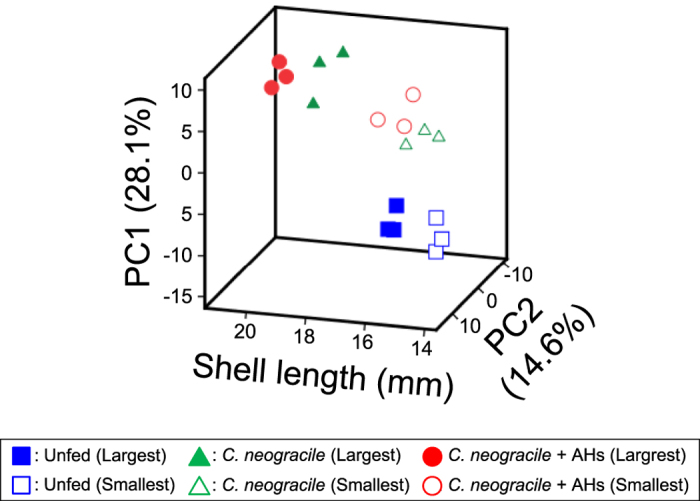
A 3D map constructed by principal component analysis (PCA) of metabolome data and shell length. The x-axis indicates the PC1 distance (%), the y-axis indicates the PC2 distance (%), and the z-axis indicates shell length (mm). See the text for a description of the PCA.

As with the heat map, the 3D map constructed by PCA of metabolome data and shell length clearly indicated the difference between the no-diet groups and the groups fed *C. neogracile* (Fig. 4). Furthermore, there was a clear separation between the maximum weights of clams in the groups given *C. neogracile* and those of the groups given AHs in addition to *C. neogracile* (Fig. 4). Similarly, there was a clear separation between the maximum- and minimum-weight clams in the no-diet groups (Fig. 4). However, PCA failed to show a clear separation between the maximum- or minimum-weight clams in the groups given *C. neogracile* and the groups given AHs in addition to *C. neogracile* (Fig. 4).

### Metabolic changes in clams under different rearing conditions

**Figure 5:**
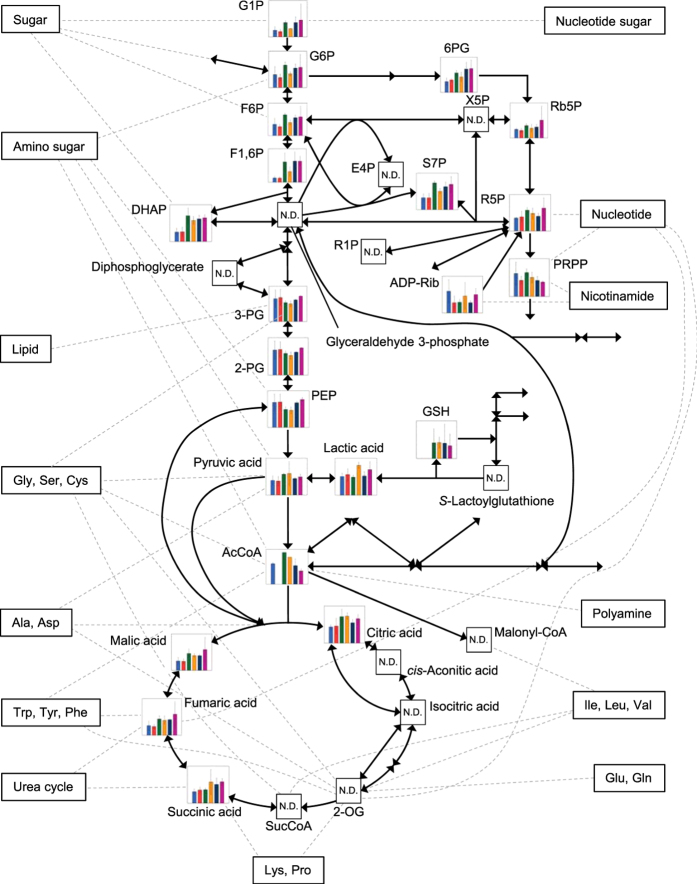
Metabolome data map of the glycolysis/glycogenesis pathways and the TCA cycle in *R. philippinarum* under different rearing conditions. Each bar represents the relative amount of a metabolite for the no-diet groups (largest clam, blue; smallest clam, bright red), the groups given *C. neogracile* only (largest clam, green; smallest clam, orange), and the groups given *C. neogracile* plus AHs (4 mg/mL) (largest clam, navy blue; smallest clam, magenta). All metabolic data are means ± SD of triplicate samples.

To provide a comprehensive and panoramic view of our results, the metabolites detected by means of CE-TOFMS were mapped onto metabolic pathways (see Supplementary Fig. S1). Throughout the metabolic pathways a large difference is evident between the starvation state of the no-diet groups and the satiation state of the groups given a specific diet (see Supplementary Fig. S1). However, at first glance there were no significant differences in the levels of metabolites between the groups given *C. neogracile* and the groups given AHs in addition to *C. neogracile* (see Supplementary Fig. S1). To find a metabolic signature in *R. philippinarum* stimulated by AHs, therefore, we tried to detect a between-group difference in the relative amounts of metabolites. As a result, we observed differences in the levels of metabolites of glycolysis/glycogenesis (Fig. 5) and amino acid metabolism (see Supplementary Appendixes S1, S2, S3) between maximum-weight clams of the groups given only *C. neogracile* and those of the groups given AHs in addition to *C. neogracile*. In particular, the level of pyruvic acid was higher in the groups given *C. neogracile* than in the groups given AHs in addition to *C. neogracile* (see Supplementary Appendixes S2, S3), whereas levels of phosphoenolpyruvic acid and acetylcholine were lower in the groups given *C. neogracile* than in the groups given AHs in addition to *C. neogracile* (see Supplementary Appendixes S2, S3). Additional detailed information for the comparison between groups is presented in Supplementary Appendixes S1, S2, and S3.

## Discussion

Our results provide several novel insights into the culture of *R. philippinarum* and conservation of the clam resource in the field. First, we demonstrated the growth-promoting effect of AHs on clams, and established a more efficient way of feeding (Figs 1 and 2). Specifically, the effective added amount of AHs is lower than that previously reported for glucose^14^, and the acidic polysaccharide alginate, known as a dietary fiber, has little stimulating effect on bacterial growth in the rearing water compared with glucose, which is generally known as an energy source. Furthermore, the alginate used in this study is food-additive grade, the same as in our previous study^20^, and its availability ensures a reliable supply of a safe diet for clams at low cost to the aquaculture industry. Thus, AHs can have a noteworthy effect on clam culture from a practical perspective.

Second, our study demonstrates a large-scale metabolic profiling of *R. philippinarum* reared under several conditions (see Supplementary Fig. S1). The metabolic pathways overall suggest that each state of starvation, food satiation, and sexual maturation has a characteristic metabolite pattern. The heat map clearly shows a difference between the no-diet groups and the groups given any diet, but it did not show a clear distinction between the groups on the two diets (*C. neogracile* and *C. neogracile* + AHs) (Fig. 3). In particular, a hierarchical cluster analysis clearly showed the difference of metabolite pattern in clams between starvation and food satiation (Fig. 3, cluster A and B). On the other hand, the 3D map constructed on the basis of the PCA of the metabolome data and shell length clearly showed a difference between the no-diet groups and the groups given *C. neogracile* and between maximum-weight clams of the groups given *C. neogracile* and those of the groups given AHs in addition to *C. neogracile* (Fig. 4). Statistical analysis, furthermore, revealed these characteristics in glycolysis/glycogenesis (Fig. 5, see Supplementary Appendixes S1, S2, S3) and in the amino acid metabolism involved in glycogenesis (see Supplementary Appendixes S1, S2, S3). It is believed that the no-diet groups consumed a substantial percentage of their stored glycogen because concentrations of glucose 1-phosphate and glucose 6-phosphate, which are well known intermediate metabolites of glycogen metabolism, were significantly reduced relative to the groups given any diet (see Supplementary Appendix S2). Downstream metabolites in the glycolysis pathway such as pyruvic acid also showed downregulation. In addition, the roundness index and weight of soft tissue of the no-diet groups were significantly lower than those of the groups given any diet (Table 1). Unfortunately, we could not analyze the glycogen content in clams because whole clams were used for the metabolomics analysis, but several studies have indicated that glycogen content in clams is positively correlated with physiological condition^21^ and the roundness index^15^. On the other hand, the metabolic pattern for glucogenic amino acids such as glycine, serine, threonine, and tryptophan in the no-diet groups differed from the other test groups. In particular, the concentration of glycine was significantly lower than that in other test groups (see Supplementary Appendixes S2, S3). Moreover, these characteristics found in the no-diet groups were more likely to be observed in the smallest clams rather than the largest. Consequently, our results suggest that the starvation state of the no-diet groups induces utilization of glycogen first through glycolysis, and then utilization of glucogenic amino acids through glycogenesis in order to compensate for the lack of an energy source. There were no notable changes in levels of glycerol or lactic acid, known sources for glycogenesis (see Supplementary Appendixes S1, S2).

Perhaps the most interesting result of our study is the growth-promoting effect of AHs on *R. philippinarum* (Figs 1 and 2, Table 1). Although the growth of clams was dramatically promoted by adding AHs to a diet of *C. neogracile* (Figs 1 and 2, Table 1), a heat map did not reveal any fundamental difference between maximum-weight clams of the groups given only *C. neogracile* and those given AHs in addition to *C. neogracile* (Fig. 3). In these circumstances, the only significant difference between these groups was the levels of phosphoenolpyruvic acid and pyruvic acid involved in glycolysis/glycogenesis (see Supplementary Appendixes S1, S2, S3). The level of phosphoenolpyruvic acid in the groups given *C. neogracile* was lower than that in the groups given AHs in addition to *C. neogracile* (see Supplementary Appendixes S1, S2, S3), whereas the levels of pyruvic acid and acetyl CoA were higher (see Supplementary Appendixes S1, S2, Fig. 5). These results suggest that, in the groups given only *C. neogracile*, carbohydrate was metabolized by glycolysis to pyruvic acid via phosphoenolpyruvic acid, and then ATP was produced by acetyl CoA converted from pyruvic acid through the TCA cycle and the respiratory chain.

Growth of *R. philippinarum* has a close relationship with maturation. In other words, clams in early stage of the development utilize energy resources on the growth, but clams in late stage of the development utilize energy resources on the maturation in addition to the growth^22^. Therefore, difference of maturity index between experimental groups implies different stage of the development. In the present study, we also found that shell length, roundness index, and maturity index were significantly higher in the groups given *C. neogracile* than in the no-diet groups (Table 1, *P* < 0.05), and were significantly lower than in the groups given AHs in addition to *C. neogracile* (Table 1, *P* < 0.05). Thus, it appears that clams in the groups given *C. neogracile* were in a growth stage with an adequate amount of carbohydrate resources. In contrast, shell length, roundness index, and maturity index were highest in the groups given AHs in addition to *C. neogracile* (Table 1, *P* < 0.05). In addition, the level of phosphoenolpyruvic acid in the groups given AHs in addition to *C. neogracile* was higher than in the groups given only *C. neogracile* (see Supplementary Appendixes S1, S2), but the levels of pyruvic acid and acetyl CoA were lower than in the groups given only *C. neogracile* (see Supplementary Appendixes S1, S2, Fig. 5). Furthermore, the levels of glucogenic amino acids such as glycine were the highest of all test groups in the groups given AHs in addition to *C. neogracile* (see Supplementary Appendixes S1, S2). This trend is attributable to the downregulation of glycolysis because of the decrease in the level of pyruvic acid, a known metabolic product of glucogenic amino acids. In addition, there was a pronounced tendency to observe these characteristics, found in the groups fed AHs in addition to *C. neogracile*, in the smallest clams rather than the largest. Consequently, growth and maturation in the groups given AHs in addition to *C. neogracile* proceeded at a rapid pace, and excess carbohydrate was actively utilized for the development of reproductive tissue.

We have not yet identified the mechanisms that explain the growth-promoting effect of AHs on clams, although this study demonstrated a metabolic signature in *R. philippinarum* stimulated by AHs. Yamasaki *et al*. demonstrated that growth of the green alga *Chlamydomonas reinhardtii* was significantly promoted by an alginate oligomer mixture (AOM) prepared by enzymatic degradation, whereas an AOM prepared by acid hydrolysis and other saccharides did not affect growth of the alga^23^. It might therefore appear that the growth-promoting effect of AHs results from their stimulating an increase in *C. neogracile* biomass. However, this was not the case, as shown by the data in Supplementary Fig. S2. Another possibility is that the growth-promoting effect of AHs results from the broad bioactivity of alginate, as shown in previous studies^16^,^24^,^25^,^26^. If this is true, the question arises as to how the clams take up AHs dissolved in seawater. Jǿrgensen reported that uptake of dissolved organic matter (DOM) such as amino acids by mussels occurs through epidermal tissue located in the mantle and gills^27^. Recently, Yamada *et al*. reported a method for imaging glucose uptake into living mammalian cells using a fluorescent D-glucose (2-NBDG) as a tracer^28^. We tried to detect glucose uptake in the clams using 2-NBDG. Our results indicated that much of the 2-NBDG was immediately taken up through the incurrent siphon and accumulated in the alimentary canal (see Supplementary Fig. S3). This suggests that much of the AHs were immediately taken up through the incurrent siphon and absorbed from the alimentary canal, and thus affected the growth of *R. philippinarum* via the bioactivity of the alginate rather than as an energy source. From another perspective, Amio *et al*. found that the density of the spirochete *Cristispira* decreased within a few hours when clams were exposed to the air (i.e. stressful conditions)^29^. Furthermore, they reported that the density of *Cristispira* in the crystalline style, which is in the alimentary canal of clams, provides a useful estimate of *R. philippinarum* vitality^30^. Thus, the growth-promoting effect of AHs on *R. philippinarum* can potentially be explained by the bioactivity of alginate on the growth of *Cristispira* in the crystalline style of the clams.

Our results demonstrate that alginate hydrolysates (AHs) can dramatically promote the growth of the Manila clam *R. philippinarum*. Thus, the utilization of AHs in the rearing of clams may be an inexpensive way to shorten the rearing time of *R. philippinarum*. Moreover, the entire metabolic pathway suggests that each state of starvation, food satiation, and sexual maturation has a characteristic pattern, and excess carbohydrate in the groups given AHs in addition to *C. neogracile* was actively utilized for the development of reproductive tissue. Hence, metabolomics can be used to evaluate the growth condition of *R. philippinarum* in a comprehensive way, and this approach is important for not only the development of a mass culture method for the clam but also for the conservation of the clam resource in the field. In particular, metabolomics targeted at clams widely distributed in nature will be an important challenge, and results will have potential use in the evaluation of dietary condition, water quality, and environmental changes.

## Methods

### Preparation of alginate hydrolysates (AHs)

We prepared AHs using sodium alginate (SKAT-ULV, KIMICA Co., Tokyo, Japan) in accordance with previous studies^20^,^23^. Samples of AHs were stored at −30 °C until use. Yamasaki *et al*. reported that the primary oligomers in AHs were a monomer (176 Da), a dimer (352 Da), and a trimer (528 Da), with no significant quantities of larger oligomers^23^.

### Microalgal diet and growth conditions

The bacillariophyte *Chaetoceros neogracile* was grown in 30-L tanks containing 20 L filtered seawater enriched with KW21 marine alga growth medium (Daiichi Seimo Co. Ltd., Kumamoto, Japan). Aerated cultures were maintained at 20 °C under continuous light.

### Clam rearing test

This test was conducted under mean water temperature of 25 °C, and was performed in triplicate. The water in the rearing tanks and the food supply was automatically exchanged four times daily (see Supplementary Fig. S4A). Aerated, filtered (pore size, 1 μm) natural seawater with mean salinity of 30–31 was used for the test.

The rearing test was conducted using 15-L tanks equipped with containers with sand (see Supplementary Fig. S4B) containing 10 L of rearing water. For each rearing test with clams, 15 clams (initial average shell length [±SD], 15.7 ± 0.3 mm) were placed into 10 L filtered seawater (i.e. no-diet), a 10-L suspension of *C. neogracile* (80,000 cells/mL), or a 10-L suspension of *C. neogracile* (80,000 cells/mL) with AHs added at 4 mg/mL, and reared for 30 d. The pH of the water was checked every day when the water was changed; there were no significant pH changes in any of the rearing tests. In addition, all containers were cleaned every ten days to prevent bacterial growth and to maintain clam survival. After the rearing test, all clams in each tank were placed into 10 L filtered seawater and incubated for 24 h without addition of any diet to allow the clams to purge their digestive systems. After 24 h, shell length, shell height, shell width, and weight of soft tissue were measured and the roundness index was calculated as follows:


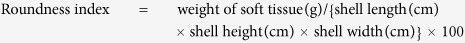


In addition, a maturity index was evaluated by visual observation (see Supplementary Fig. S5) according to a previous study^31^. Clams in each tank were then ranked in order of their soft tissue weight, and the soft tissues of the largest and smallest clams in each tank in terms of soft tissue weight were stored at −80 °C until metabolite extraction.

### Effect of AHs on the growth of microalgal food

To determine whether AHs affected the growth of *C. neogracile*, culture experiments were conducted in 500-mL beakers containing 500 mL of medium. As a test group, a 500-mL suspension of *C. neogracile* (80,000 cells/mL) with AHs at 4 mg/mL was incubated for 24 h. As a control, a 500-mL suspension of *C. neogracile* (80,000 cells/mL) without added AHs was incubated for 24 h. There were three replicate beakers for each treatment, and aerated cultures were maintained at 25 °C in the dark. *Chaetoceros neogracile* cells were counted microscopically in 1000-μL subsamples collected 0, 2, 4, 6 and 24 h after the start of the experiment. Throughout all experiments, cell counting was repeated five times for each sample.

### Metabolite extraction

Clam metabolites were extracted from 50 mg of soft tissue by addition of 1.5 mL of 50% acetonitrile solution (v/v) using a homogenizer in accordance with a previous study^32^. The extract solution was centrifuged for 5 min at 2300 × *g*, 4 °C, and the supernatant was subjected to ultrafiltration.

Ultrafiltration was performed by using a centrifugal filter device (UltrafreeMC-PLHCC; Human Metabolome Technologies, Yamagata, Japan) with a regenerated cellulose membrane (molecular weight cutoff [MWCO], 5 kDa) and was carried out by using two centrifugal filter devices per sample. An aliquot of 400 μL of extract solution was subjected to ultrafiltration for 2 h at 9100 × *g*, 4 °C. The permeate fraction (MWCO <10 kDa) was then dried and suspended in 50 μL of Milli-Q water.

### Analysis of clam metabolites

All analyses of metabolites were performed by using a capillary electrophoresis time-of-flight mass spectrometry (CE-TOFMS) system (Agilent 7100 CE and 6210 TOFMS system, Agilent Technologies, Santa Clara, CA, USA) according to previous studies^33^,^34^,^35^,^36^. Metabolites were analyzed using a fused silica capillary (i.d. 50 μm × 80 cm), with commercial electrophoresis buffer as run and rinse buffer (product number H3301-1001 for cation analysis and I3302–1023 for anion analysis; Human Metabolome Technologies). The sample was injected at a pressure of 50 mbar for 10 sec for the cation analysis, and at 50 mbar for 25 sec for the anion analysis. The TOFMS detected mass-to-charge ratios (*m/z*) of 50–1000. In addition, analysis of clam metabolites was carried out by Human Metabolome Technology Inc. (Yamagata, Japan), and lipids in the samples were not removed because those did not affect CE-TOFMS analysis.

### Peak annotation and pathway analysis

To obtain information for the analytes with reference to *m/z*, migration time (MT), and peak area, the peaks detected by use of CE-TOFMS were extracted using automatic integration software (MasterHands ver.2.13.0.8.h, Keio University, Tsuruoka, Japan) according to a previous study^37^. After excluding adduct ions and fragment ions included in signal peaks, the remaining peaks were collated with metabolites registered in the Human Metabolome Technologies metabolite database (HMDB), on the basis of their MT and *m/z* values. The acceptable error range of peak annotation was set at ±0.5 min for MT and ±10 ppm for *m/z*. Furthermore, peak areas of major metabolites including amino acids, organic acids, sugar phosphates, and nucleic acids, were normalized by using an internal standard (200 μM), and then the corrected values of these metabolites were used in the quantification. In addition, principal component analysis (PCA) was carried out using SampleStat software (Human Metabolome Technologies), and hierarchical cluster analysis (HCA) and formulation of a heat map were performed by using PeakStat software (Human Metabolome Technologies). Metabolic pathway maps were drawn based on enzymes derived from humans by utilizing the network visualization and analysis tool Visualization and Analysis of Networks containing Experimental Data (VANTED, http://vanted.ipk-gatersleben.de/). In addition, metabolite annotations from both the HMDB and the Kyoto Encyclopedia of Genes and Genomes (KEGG) databases were used to link the metabolites to metabolic pathways.

### Imaging clam glucose uptake

To visualize glucose uptake into the clams, we used a fluorescent D-glucose derivative, 2-[N-(7-nitrobenz-2-oxa-1,3-diazol-4-yl) amino]-2-deoxy-D-glucose (2-NBDG; Wako Pure Chemical Industries Ltd., Osaka, Japan), as a tracer. Clams were loaded with 1000 μL of filtered seawater containing 200 μM of 2-NBDG and incubated for 2 min, then washed twice with 1000 μL of fresh filtered seawater. Live clams were then observed by using a fluorescent microscope (Eclipse Ti-U, Nikon, Tokyo, Japan) with excitation wavelength of 465–495 nm and absorption wavelength of 515–555 nm.

### Statistical analysis

The data from the clam rearing test were analyzed by one-way analysis of variance (ANOVA) and then tested by using Tukey’s post hoc test. In addition, the data from the metabolite analysis were analyzed by using Welch’s test. The analysis was performed using SPSS for Windows (SPSS version 19.0; SPSS, Inc., Chicago, IL, USA). A significance level of *P* < 0.05 was used for the test.

## Additional Information

**How to cite this article**: Yamasaki, Y. *et al*. A metabolic profile in *Ruditapes philippinarum* associated with growth-promoting effects of alginate hydrolysates. *Sci. Rep.* **6**, 29923; doi: 10.1038/srep29923 (2016).

## Supplementary Material

Supplementary Information

## Figures and Tables

**Table 1:** Shell size, soft tissue wet-weight, roundness index, and maturity index of *Ruditapes philippinarum* after 30-day cultures 1) without food, 2) with only *Chaetoceros neogracile* (80,000 cells/mL), and 3) with *C. neogracile* (80,000 cells/mL) supplemented with alginate hydrolysates (AHs) at 4 mg/mL.

	**Unfed control**	***C. neogracile***	***C. neogracile*** **with AHs (4 mg/mL)**
Shell height (mm)	10.9 ± 0.00385^a^	11.9 ± 0.117^b^	12.5 ± 0.220^c^
Shell width (mm)	6.00 ± 0.0402^a^	6.70 ± 0.0769^b^	6.94 ± 0.137^c^
Soft tissue (mg wet weight)	145 ± 5.27^a^	267 ± 12.5^b^	341 ± 13.9^c^
Roundness index^1^	14.1 ± 0.441^a^	19.2 ± 0.919^b^	21.1 ± 0.628^c^
Maturity index^2^	0.0556 ± 0.0.0192^a^	0.400 ± 0^b^	0.633 ± 0.0667^c^

^1^Roundness index was calculated by the method described in the text.

^2^Maturity index was determined by visual observation of gonad.

Data are means ± SD of triplicate measurements. For each property, values with different letters are significantly different at *P* < 0.05 (one-way analysis of variance (ANOVA) and then tested by using Tukey’s post hoc test).
