# Corrigendum: A metabolic profile in *Ruditapes philippinarum* associated with growth-promoting effects of alginate hydrolysates

**DOI:** 10.1038/srep32829

**Published:** 2016-09-12

**Authors:** Yasuhiro Yamasaki, Shigeru Taga, Masanobu Kishioka, Shuichi Kawano

Scientific Reports 6: Article number: 2992310.1038/srep29923; published online: 07 20 2016; updated: 09 12 2016

In the original version of this Article, all instances of “4 mg/L” were incorrectly given as “4 mg/mL”. This error has now been corrected in the PDF and HTML versions of the Article.

