# Supplementary material for: A metabolic profile in Ruditapes philippinarum associated with growth-promoting effects of alginate hydrolysates
Source: Sci Rep. 2016 Jul 20;6:29923. doi: 10.1038/srep29923 (PMC4951710; doi:10.1038/srep29923)
Supplement: Supplementary Information [file srep29923-s1.pdf]

Supplementary Information

A metabolic profile in *Ruditapes philippinarum* associated with growth-promoting effects of alginate hydrolysates

Yasuhiro Yamasaki[1,*], Shigeru Taga[2], Masanobu Kishioka[2], & Shuichi Kawano[3]

[1]Laboratory of Environmental Biology, Department of Applied Aquabiology, National Fisheries University, Yamaguchi, Japan; [2]Yamaguchi Prefectural Fisheries Research Center, Yamaguchi, Japan; [3]Graduate School of Informatics and Engineering, The University of Electro-Communications, Tokyo, Japan

Correspondence and requests for materials should be addressed to Y.Y.
(email: yamasaky@fish-u.ac.jp)

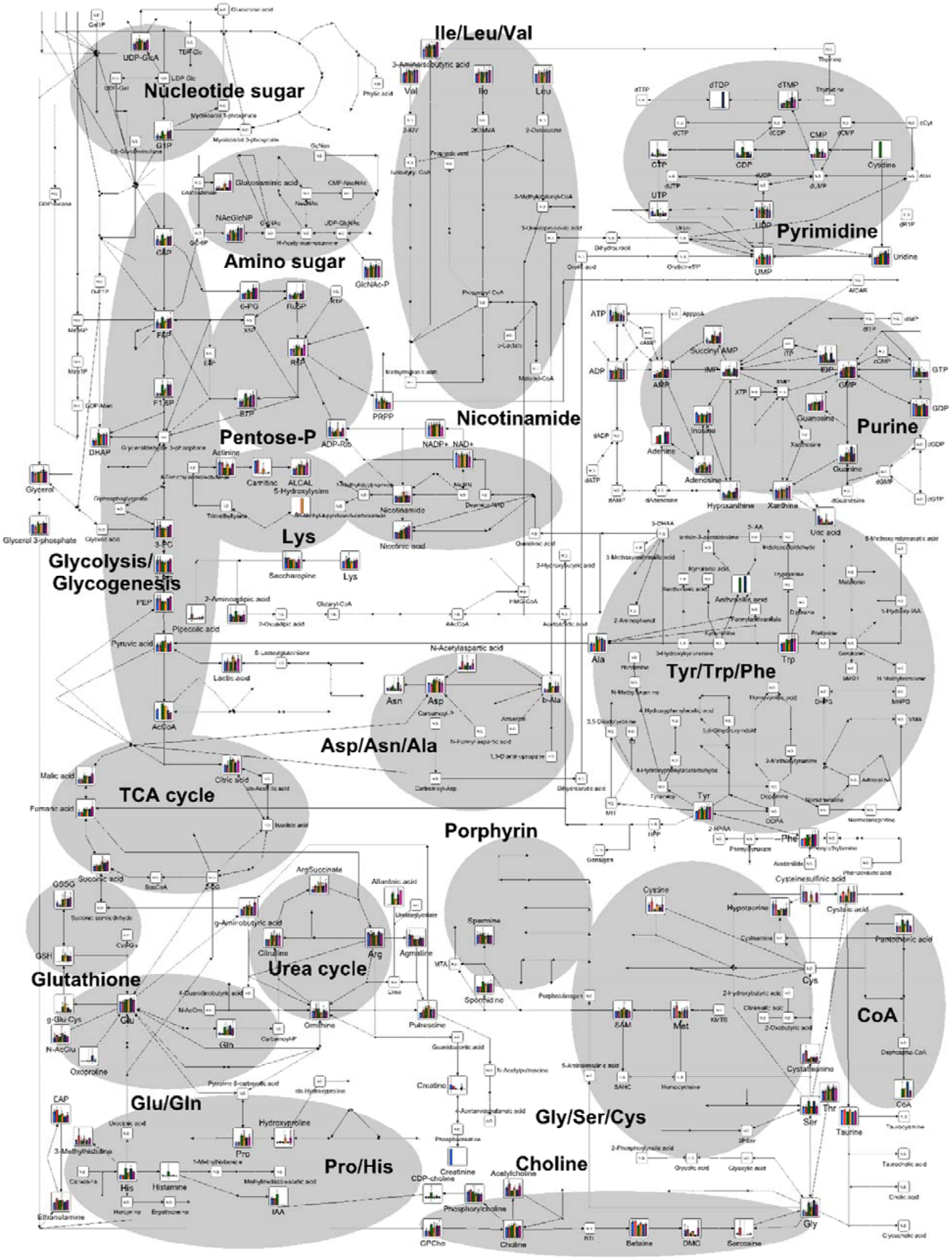

Figure S1. Metabolome data map of all metabolic pathways in *R. philippinarum* under the different rearing conditions. Each bar represents the amount of a metabolite normalized to an internal standard for the different diet treatments: no-diet (largest clam, blue; smallest clam, bright red), *C. neogracile* only (largest clam, green; smallest clam, orange), and *C. neogracile* plus AHs (4 mg/mL) (largest clam, navy-blue; smallest clam, magenta). All metabolic data are mean ± SD of triplicate samples.

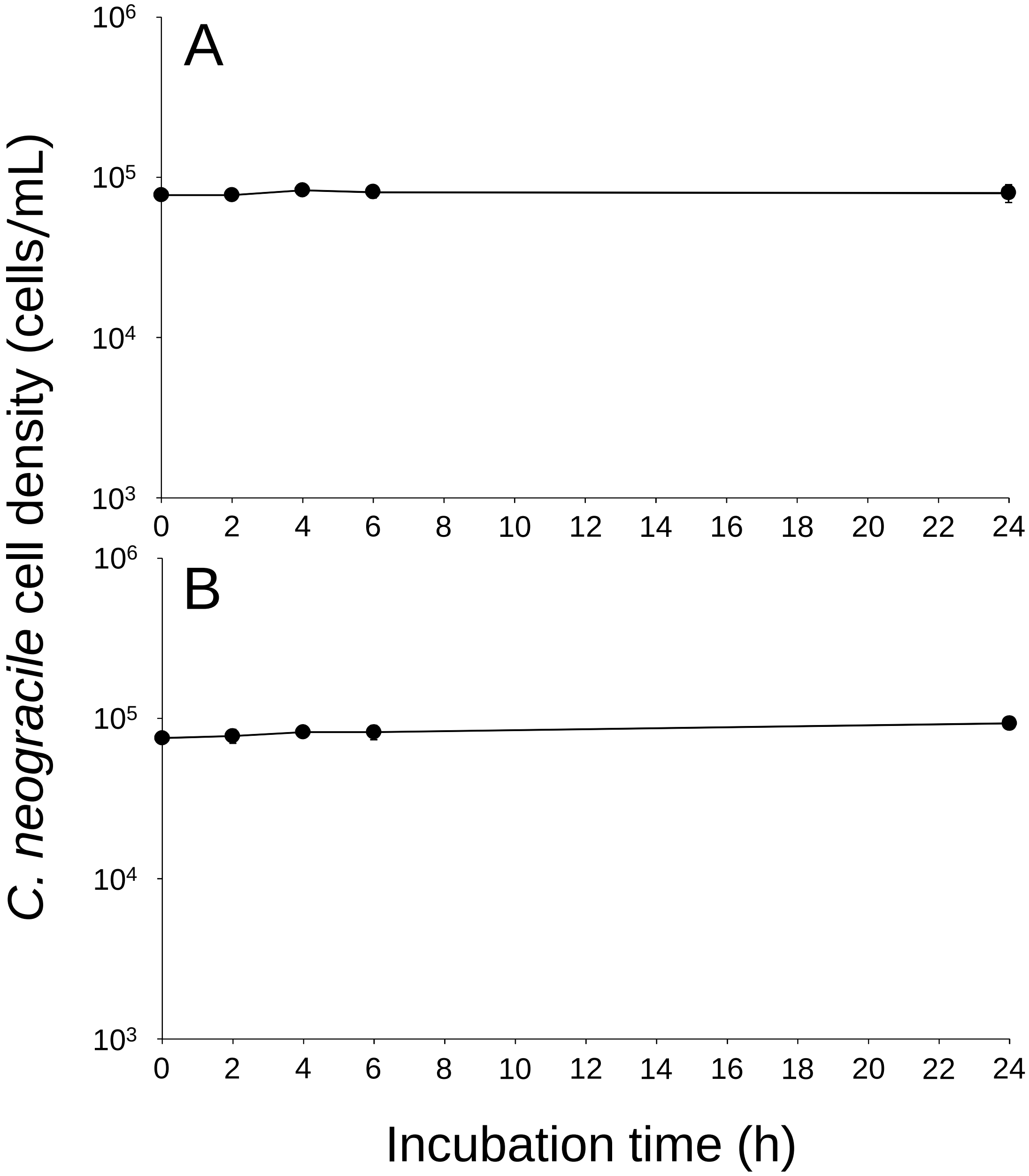

Figure S2. Effect of AHs on the growth of *C. neogracile*. (A) Cell density of *Chaetoceros neogracile* without added AHs at 0, 2, 4, 6, and 24 hours after the start of the experiment. (B) Cell density of *Chaetoceros neogracile* with AHs added at 4 mg/mL at 0, 2, 4, 6, and 24 hours after the start of the experiment. Data are means ± SD (cells/mL) of triplicate measurements.

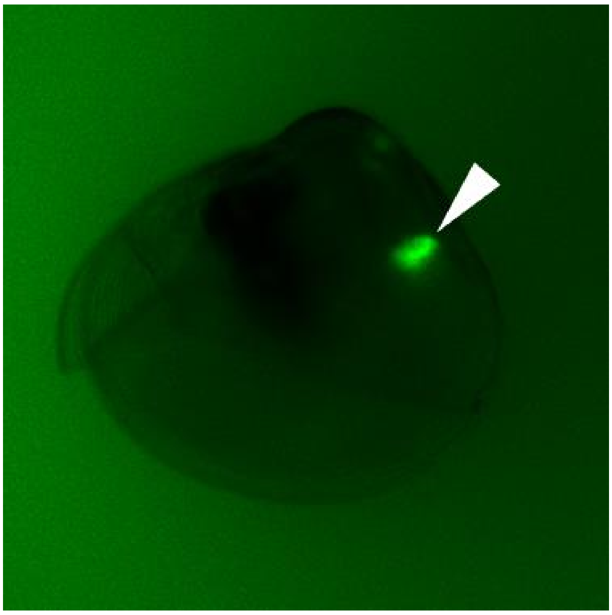

Figure S3. Image of glucose uptake into a clam using the fluorescent D-glucose derivative 2-[N-(7-nitrobenz-2-oxa-1,3-diazol-4-yl)amino]-2-deoxy- D-glucose (2-NBDG) as a tracer. The fluorescent image was taken at wavelength 540 nm after loading 1000 μL of filtered seawater with 200 μM 2-NBDG.

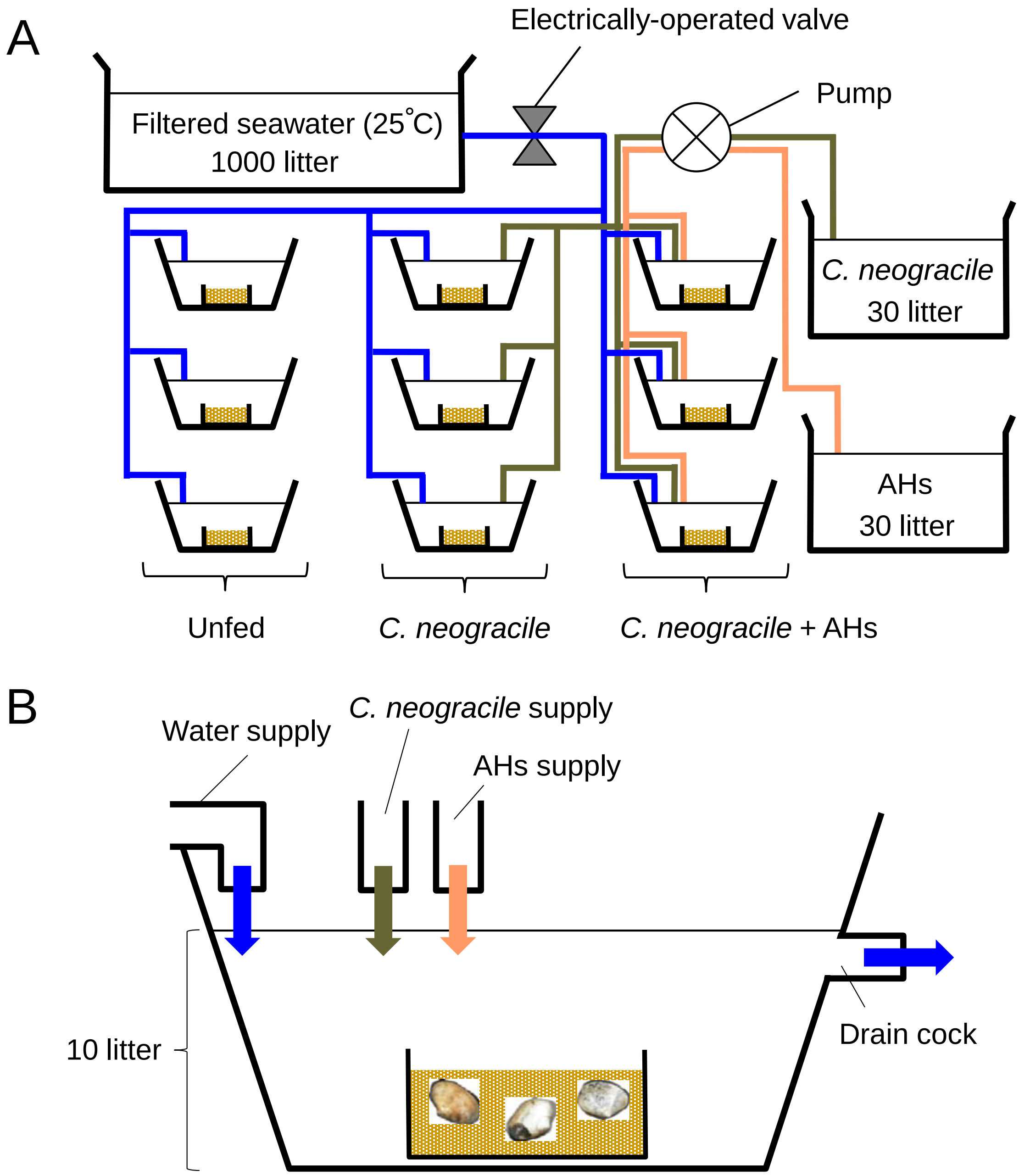

Figure S4. Schematics of the rearing experiment. (A) Automatic food distribution system used in the rearing experiment. (B) Cross section of a 15-L tank used in the rearing experiment.

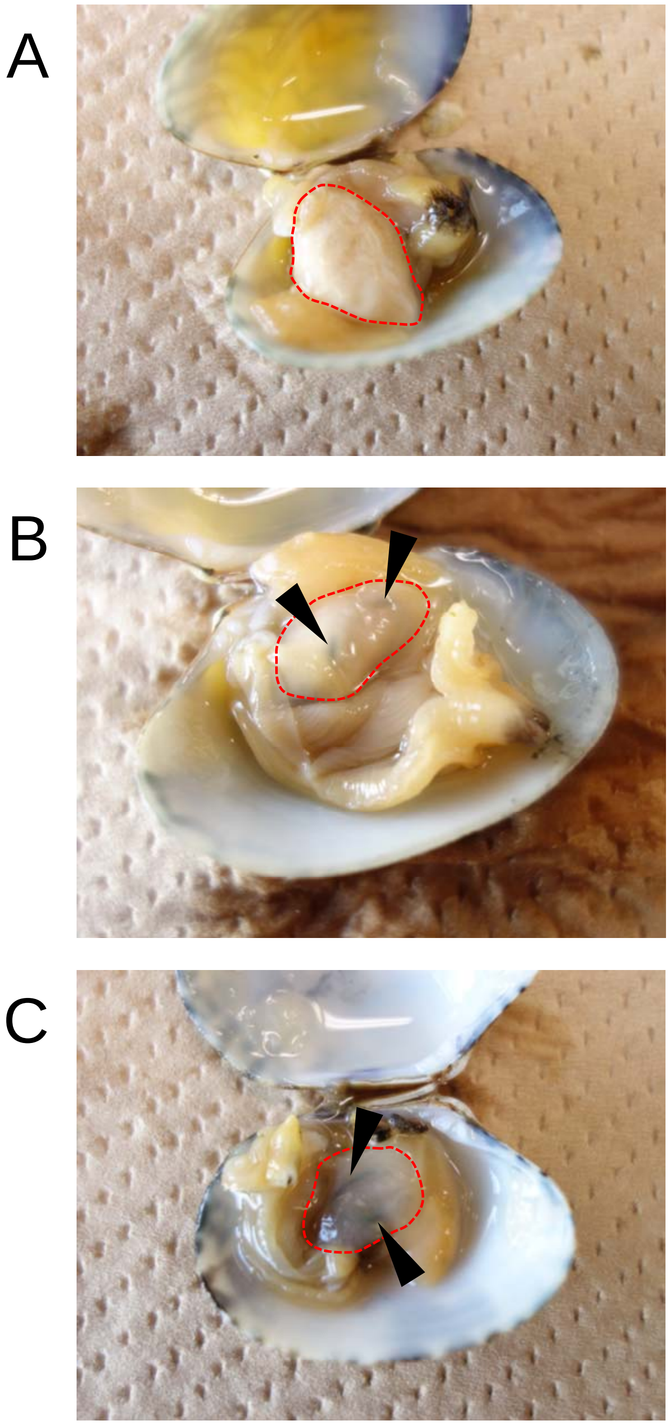

Figure S5. Photographs of *R. philippinarum* gonad under various conditions. (A) Fully mature gonad, obscuring the alimentary canal. (B) Partially developed gonads. (C) Immature clam; the alimentary canal alone is visible, without gonad. Red dashed lines indicate the gonad in *R. philippinarum* and arrow indicates the alimentary canal.

Appendix S1 Relative peak areas of metabolites detected by using CE-TOFMS after normalization to an internal standard. Values are for the largest and smallest clams in each group.

Compound name or estimated molecular formula	Relative area											
	Unfed (largest)		Unfed (smallest)		*C. neogracile* (largest)		*C. neogracile* (smallest)		*C. neogracile* + AHs (largest)		*C. neogracile* + AHs (smallest)	
	Mean	S.D.	Mean	S.D.	Mean	S.D.	Mean	S.D.	Mean	S.D.	Mean	S.D.
2,3-Diaminopropionic acid	1.6E-03	5.4E-05	1.3E-03	2.1E-04	5.4E-04	1.1E-04	8.4E-04	6.7E-05	3.8E-04	1.3E-04	8.6E-04	3.0E-04
2,4-Diaminobutyric acid	2.4E-04	1.3E-05	2.2E-04	9.3E-06	5.5E-04	1.1E-04	3.4E-04	2.7E-04	6.1E-04	6.0E-05	4.6E-04	2.0E-04
2,6-Diaminopimelic acid	2.9E-04	2.1E-05	4.8E-04	3.6E-04	1.8E-03	1.6E-03	2.0E-03	2.6E-03	5.8E-04	3.3E-04	7.5E-04	3.6E-04
2-Amino-3-phosphonopropionic acid	4.6E-03	2.4E-03	6.5E-03	2.8E-03	1.8E-03	5.7E-04	1.5E-03	1.2E-04	1.7E-03	1.0E-03	1.3E-03	1.9E-04
2-Aminoadipic acid	2.0E-02	1.8E-02	1.2E-02	2.9E-03	2.7E-02	1.2E-02	1.6E-02	9.9E-03	1.5E-02	3.4E-03	1.8E-02	1.0E-02
2-Aminobutyric acid	2.5E-02	6.6E-03	2.5E-02	1.1E-02	3.3E-02	7.0E-03	2.6E-02	9.1E-03	2.5E-02	2.0E-03	2.5E-02	6.4E-03
2-Aminoethylphosphonic acid	1.4E-02	2.8E-03	1.5E-02	2.1E-03	4.9E-03	1.0E-03	5.2E-03	1.8E-03	3.6E-03	3.2E-04	3.6E-03	7.6E-04
2-Deoxyglucose 6-phosphate	5.3E-05	2.2E-05	4.7E-05	3.5E-05	2.5E-04	6.4E-05	2.2E-04	3.3E-05	2.9E-04	1.1E-04	2.5E-04	8.1E-05
2-Methylserine	3.3E-03	5.9E-04	4.1E-03	1.3E-03	3.7E-03	3.1E-04	2.9E-03	1.1E-03	3.6E-03	1.2E-03	2.8E-03	8.4E-04
2-Phosphoglyceric acid	4.5E-04	1.6E-04	4.6E-04	1.4E-04	3.9E-04	4.2E-05	3.6E-04	6.4E-05	4.2E-04	1.5E-05	4.9E-04	1.1E-05
3-Aminoisobutyric acid	2.5E-03	5.9E-04	3.3E-03	8.4E-04	3.9E-03	7.5E-04	3.6E-03	1.3E-03	3.9E-03	9.5E-04	4.6E-03	2.8E-04
3-Hydroxy-3-methylglutaric acid	5.8E-05	9.9E-06	4.5E-05	N.A.	8.5E-05	2.9E-05	1.9E-04	1.3E-04	5.3E-05	1.6E-06	9.3E-05	5.4E-05
3-Methylhistidine	1.0E-03	9.9E-05	2.0E-03	1.6E-03	8.8E-04	4.8E-04	1.2E-03	8.0E-04	8.3E-04	3.5E-04	1.3E-03	2.4E-04
3-Phosphoglyceric acid	3.7E-03	1.5E-03	3.9E-03	1.3E-03	3.1E-03	3.3E-04	2.9E-03	4.9E-04	3.6E-03	8.5E-05	4.2E-03	1.4E-04
5-Hydroxylysine	N.D.	N.A.	1.7E-04	N.A.	N.D.	N.A.	2.4E-03	N.A.	N.D.	N.A.	N.D.	N.A.
5-Oxoproline	7.6E-04	4.5E-04	6.0E-04	1.5E-04	1.9E-03	5.4E-04	1.5E-03	8.1E-04	2.1E-02	3.1E-02	4.6E-03	6.8E-03
6-Phosphogluconic acid	3.3E-03	1.1E-03	3.8E-03	5.3E-04	5.9E-03	8.0E-04	4.8E-03	1.4E-03	7.1E-03	2.1E-03	7.3E-03	2.6E-03

ADMA	5.8E-04	7.2E-05	5.1E-04	7.0E-05	8.8E-04	1.4E-04	8.4E-04	3.8E-04	9.0E-04	3.2E-04	1.1E-03	4.8E-04
ADP	4.8E-02	7.3E-03	4.3E-02	7.6E-03	5.2E-02	1.9E-02	5.9E-02	5.2E-03	5.7E-02	5.0E-03	5.4E-02	3.5E-02
ADP-glucose GDP-fucose	2.1E-04	1.2E-05	2.5E-04	3.3E-05	2.5E-04	2.8E-05	2.5E-04	4.2E-05	2.5E-04	3.5E-05	3.0E-04	1.4E-05
ADP-ribose	2.0E-04	1.1E-04	9.6E-05	6.4E-05	1.0E-04	2.7E-05	1.6E-04	1.2E-04	9.6E-05	1.9E-05	1.7E-04	5.2E-05
AMP	1.2E-02	1.1E-02	1.9E-02	1.0E-02	2.3E-02	1.4E-02	3.8E-02	2.7E-02	3.4E-02	1.2E-02	3.4E-02	7.8E-03
ATP	5.2E-02	2.1E-02	2.8E-02	1.9E-02	4.6E-02	1.9E-02	3.9E-02	3.4E-02	3.1E-02	9.4E-03	2.8E-02	2.3E-02
Acetyl CoA_divalent	2.8E-05	1.4E-06	N.D.	N.A.	4.4E-05	N.A.	3.7E-05	N.A.	2.6E-05	9.2E-06	1.8E-05	2.8E-06
Acetylcholine	3.2E-03	8.6E-04	4.5E-03	1.4E-03	2.2E-03	1.1E-04	3.2E-03	6.8E-04	2.4E-03	7.0E-05	3.8E-03	8.1E-04
Adenine	N.D.	N.A.	7.9E-05	N.A.	9.1E-05	N.A.	N.D.	N.A.	1.3E-04	4.2E-05	1.3E-04	N.A.
Adenosine	2.0E-03	1.0E-03	3.0E-03	1.2E-03	3.3E-03	6.0E-04	4.3E-03	2.5E-03	2.6E-03	9.1E-04	3.4E-03	8.7E-04
Adenylosuccinic acid	8.2E-05	4.4E-05	5.0E-05	1.2E-05	1.6E-04	1.9E-05	1.6E-04	9.1E-05	1.4E-04	3.9E-05	2.1E-04	1.1E-04
Adipic acid	8.2E-05	N.A.	7.6E-05	N.A.	3.9E-04	2.7E-04	3.6E-04	1.9E-04	3.0E-04	1.9E-04	5.9E-04	2.5E-04
Agmatine	3.4E-03	1.3E-03	3.0E-03	3.8E-04	2.1E-03	8.2E-04	1.9E-03	1.3E-03	2.3E-03	5.3E-04	2.3E-03	3.6E-04
Ala-Ala	3.1E-04	1.1E-04	3.5E-04	3.7E-05	7.2E-04	4.3E-05	4.6E-04	8.0E-05	7.8E-04	1.9E-04	6.1E-04	2.8E-04
Ala	3.2E-02	5.4E-03	3.0E-02	7.3E-03	5.9E-02	6.3E-03	5.0E-02	1.2E-02	5.2E-02	5.9E-03	4.5E-02	5.4E-03
Allantoic acid	N.D.	N.A.	N.D.	N.A.	1.9E-04	3.5E-05	9.0E-05	4.0E-06	1.7E-04	9.1E-05	2.3E-04	N.A.
Anthranilic acid	N.D.	N.A.	N.D.	N.A.	5.5E-02	N.A.	N.D.	N.A.	5.8E-02	N.A.	N.D.	N.A.
Arg	1.0E+00	1.0E-01	9.1E-01	2.3E-01	1.0E+00	1.9E-01	7.9E-01	6.3E-02	1.1E+00	6.1E-02	9.8E-01	8.5E-02
Argininosuccinic acid	7.1E-04	4.0E-04	1.5E-03	4.2E-04	1.3E-03	1.1E-03	2.3E-03	1.2E-03	1.5E-03	7.0E-04	2.1E-03	9.5E-04
Ascorbate 2-glucoside	5.4E-05	1.0E-05	5.8E-05	1.6E-05	7.8E-05	2.4E-05	1.2E-04	4.7E-05	1.3E-04	3.5E-05	1.2E-04	8.6E-05
Asn	2.2E-02	8.1E-03	2.4E-02	1.3E-02	3.8E-02	2.1E-02	1.5E-02	1.3E-02	7.5E-02	4.9E-02	1.9E-02	3.0E-02
Asp	7.8E-01	5.8E-02	7.0E-01	1.0E-01	1.0E+00	1.5E-01	1.0E+00	2.7E-01	9.3E-01	1.9E-01	7.8E-01	2.0E-01
Azetidine 2-carboxylic acid	3.2E-04	1.0E-04	3.4E-04	3.8E-05	N.D.	N.A.	1.9E-04	N.A.	N.D.	N.A.	1.9E-04	6.8E-05
Betaine	4.7E-01	4.9E-02	4.9E-01	3.1E-02	3.5E-01	3.1E-02	4.2E-01	4.5E-02	3.8E-01	1.5E-02	4.1E-01	4.6E-02
Betonicine	6.1E-02	8.3E-03	6.2E-02	7.9E-03	2.7E-02	1.5E-03	3.7E-02	5.8E-03	2.7E-02	3.1E-03	3.9E-02	1.1E-02
Biopterin	2.6E-03	1.5E-03	3.3E-03	1.8E-03	2.2E-03	1.9E-03	2.1E-03	7.4E-04	1.5E-03	1.5E-03	2.6E-03	8.4E-04
Butyrylcarnitine	3.9E-04	9.5E-05	5.0E-04	5.1E-05	7.6E-04	3.1E-04	9.6E-04	1.3E-04	1.1E-03	3.2E-04	1.1E-03	5.8E-04
CDP	1.5E-04	3.4E-05	8.7E-05	3.6E-05	3.8E-04	2.0E-04	1.9E-04	7.4E-05	2.0E-04	5.7E-05	1.3E-04	9.4E-05

CDP-choline	2.8E-04	1.9E-04	3.7E-04	1.1E-04	1.3E-03	2.0E-03	3.2E-04	2.1E-04	5.5E-04	3.6E-04	3.7E-04	7.6E-05
CMP	1.4E-04	2.6E-05	1.6E-04	5.7E-05	4.7E-04	3.4E-04	2.5E-04	7.9E-05	3.2E-04	1.3E-04	2.2E-04	1.2E-05
CTP	1.1E-04	4.9E-05	4.9E-05	3.3E-05	3.2E-04	3.4E-04	1.3E-04	1.3E-04	9.2E-05	5.8E-05	7.5E-05	2.8E-05
Cadaverine	4.8E-03	1.1E-03	3.6E-03	1.8E-03	1.0E-03	4.8E-04	1.4E-03	1.7E-03	5.0E-04	3.3E-04	1.7E-03	1.9E-03
Carnitine	8.9E-02	4.9E-03	8.8E-02	9.8E-03	3.8E-03	1.1E-03	3.2E-02	4.1E-02	2.2E-03	3.2E-04	6.0E-03	3.6E-03
Choline	1.5E-02	1.8E-03	1.6E-02	3.5E-03	1.8E-02	3.2E-03	2.1E-02	2.6E-03	1.6E-02	4.1E-03	1.8E-02	9.1E-04
Citric acid	3.5E-03	4.0E-04	3.7E-03	1.7E-03	5.8E-03	1.6E-03	5.9E-03	7.3E-04	4.4E-03	7.0E-04	5.3E-03	8.9E-04
Citrulline	9.1E-04	1.4E-04	9.4E-04	1.2E-04	1.6E-03	3.6E-04	1.5E-03	8.1E-04	1.6E-03	2.8E-04	1.5E-03	4.2E-04
CoA_divalent	N.D.	N.A.	N.D.	N.A.	9.2E-05	8.1E-05	1.0E-04	N.A.	1.8E-04	N.A.	7.2E-05	3.7E-05
Creatine	5.5E-03	8.7E-03	4.0E-03	N.A.	1.1E-03	8.3E-04	2.4E-04	8.1E-06	2.8E-03	4.1E-03	3.6E-04	N.A.
Creatinine	2.3E-04	N.A.	N.D.	N.A.	N.D.	N.A.	N.D.	N.A.	N.D.	N.A.	N.D.	N.A.
Cystathionine	4.4E-03	2.8E-04	6.7E-03	3.1E-03	1.6E-03	8.0E-04	2.4E-03	8.3E-04	2.4E-03	9.3E-04	2.7E-03	1.8E-03
Cysteic acid	6.9E-03	2.3E-03	1.1E-02	9.7E-04	6.8E-03	3.0E-03	1.5E-02	2.9E-03	1.1E-02	1.7E-03	1.2E-02	6.5E-03
Cysteine glutathione disulfide	4.5E-04	2.3E-04	3.2E-04	1.3E-04	4.7E-04	1.1E-04	4.9E-04	1.3E-04	5.4E-04	2.0E-04	5.8E-04	9.9E-05
Cysteinesulfinic acid	2.8E-04	1.5E-04	2.7E-04	1.7E-05	N.D.	N.A.	2.0E-04	9.4E-05	N.D.	N.A.	2.4E-04	N.A.
Cystine	1.8E-04	6.1E-05	2.7E-04	9.5E-05	6.1E-05	N.A.	1.8E-04	3.5E-05	1.1E-04	6.5E-05	1.8E-04	2.1E-04
Cytidine	N.D.	N.A.	N.D.	N.A.	1.7E-03	N.A.	N.D.	N.A.	N.D.	N.A.	N.D.	N.A.
Cytosine	2.1E-04	N.A.	3.7E-04	N.A.	N.D.	N.A.	N.D.	N.A.	N.D.	N.A.	N.D.	N.A.
Dihydroxyacetone phosphate	1.1E-03	4.3E-04	1.1E-03	5.3E-04	3.0E-03	9.0E-04	2.5E-03	4.5E-04	2.7E-03	4.2E-04	2.8E-03	3.7E-04
Ectoine	2.6E-02	1.5E-02	3.0E-02	1.2E-02	8.1E-02	3.4E-03	5.1E-02	1.1E-02	9.3E-02	1.3E-02	7.4E-02	2.7E-02
Ethanolamine	5.6E-04	9.9E-06	8.4E-04	1.1E-04	7.2E-04	1.9E-05	7.4E-04	4.8E-04	6.2E-04	2.1E-05	7.3E-04	N.A.
Ethanolamine phosphate	4.3E-03	7.8E-04	5.0E-03	1.4E-03	2.7E-03	7.7E-04	3.4E-03	9.3E-04	2.0E-03	2.7E-04	2.6E-03	1.0E-03
Ethyl glucuronide	1.5E-03	6.7E-04	1.7E-03	7.7E-04	2.0E-04	1.4E-04	1.4E-04	4.2E-05	2.5E-04	1.6E-04	1.9E-04	4.8E-05
FAD_divalent	4.8E-04	2.9E-04	4.8E-04	8.7E-05	4.2E-04	2.3E-05	4.0E-04	1.1E-04	3.3E-04	1.2E-04	3.7E-04	6.7E-05
Formiminoglutamic acid	2.9E-04	7.3E-05	1.9E-04	1.4E-04	1.2E-04	1.3E-05	7.6E-05	8.2E-06	1.6E-04	5.8E-05	1.3E-04	3.5E-05
Fructose 1,6-diphosphate	2.1E-04	8.2E-05	2.0E-04	6.0E-05	9.7E-04	4.3E-04	5.1E-04	2.1E-04	1.0E-03	4.4E-04	1.0E-03	2.4E-04

Fructose 6-phosphate	3.4E-03	1.7E-03	2.7E-03	6.4E-04	6.2E-03	5.3E-04	4.0E-03	7.0E-04	5.3E-03	8.9E-04	5.6E-03	3.3E-03
Fumaric acid	3.1E-03	1.5E-03	2.8E-03	6.3E-04	5.2E-03	9.5E-04	4.8E-03	1.3E-03	5.0E-03	2.8E-04	6.7E-03	3.9E-03
GABA	7.1E-04	1.1E-04	9.3E-04	1.0E-04	1.1E-03	1.3E-04	1.1E-03	3.6E-04	9.5E-04	3.3E-05	1.4E-03	2.6E-04
GDP	2.4E-03	7.3E-05	2.3E-03	5.6E-04	1.9E-03	3.4E-04	2.0E-03	7.0E-04	2.2E-03	2.5E-04	1.9E-03	1.0E-03
GDP-glucose GDP-mannose GDP-galactose	4.1E-04	6.1E-05	4.6E-04	6.2E-05	7.4E-04	4.1E-04	6.1E-04	6.3E-05	4.4E-04	2.9E-05	5.4E-04	1.7E-04
GMP	3.5E-03	1.1E-03	5.5E-03	1.8E-03	6.3E-03	5.5E-04	6.2E-03	3.0E-03	6.4E-03	8.9E-04	6.1E-03	6.4E-04
GTP	1.7E-03	6.9E-04	9.2E-04	8.2E-04	1.6E-03	1.1E-03	8.7E-04	8.7E-04	8.7E-04	4.3E-04	8.7E-04	1.8E-04
Gln	1.3E-01	6.8E-02	1.1E-01	4.5E-02	5.9E-01	2.8E-01	3.6E-01	3.0E-01	6.8E-01	1.2E-01	5.0E-01	6.0E-01
Glu	8.8E-01	6.1E-02	8.7E-01	1.4E-01	1.1E+00	5.7E-02	1.1E+00	6.0E-02	1.2E+00	1.6E-01	1.1E+00	2.1E-01
Gluconic acid	5.4E-04	4.1E-05	5.3E-04	7.0E-05	7.2E-04	2.1E-04	8.5E-04	3.2E-04	8.4E-04	1.0E-04	8.8E-04	2.2E-04
Glucosaminic acid	8.5E-04	6.7E-04	9.2E-04	6.1E-04	8.0E-04	4.6E-04	2.3E-03	8.5E-04	1.2E-03	7.2E-04	3.2E-03	2.1E-03
Glucose 1-phosphate	9.1E-04	4.9E-04	7.4E-04	2.5E-04	1.9E-03	2.4E-04	1.1E-03	1.8E-05	1.9E-03	7.9E-04	2.3E-03	2.2E-03
Glucose 6-phosphate	1.6E-02	9.1E-03	1.2E-02	4.2E-03	2.7E-02	3.8E-03	1.7E-02	2.4E-03	2.3E-02	4.8E-03	2.4E-02	1.5E-02
Glutathione (GSH)	N.D.	N.A.	N.D.	N.A.	3.4E-03	3.1E-03	3.5E-03	1.2E-03	3.4E-03	4.1E-03	2.8E-03	2.6E-03
Glutathione (GSSG)_divalent	2.6E-03	1.5E-03	1.2E-03	8.3E-04	5.2E-03	3.7E-03	5.6E-03	5.6E-03	6.0E-03	1.5E-03	6.6E-03	3.9E-03
Gly	3.3E-02	8.6E-03	2.4E-02	1.0E-02	8.2E-02	1.3E-02	5.7E-02	2.6E-02	9.6E-02	4.1E-03	7.1E-02	1.7E-02
Glycerol	1.1E-01	5.2E-02	9.0E-02	1.6E-02	1.0E-01	6.1E-03	1.1E-01	1.5E-02	1.3E-01	2.9E-02	1.1E-01	1.2E-02
Glycerol 3-phosphate	2.0E-03	2.6E-04	1.6E-03	4.9E-04	2.6E-03	4.0E-04	2.2E-03	9.8E-05	2.5E-03	3.2E-04	2.6E-03	1.2E-03
Glycerophosphocholine	1.7E-02	8.4E-03	1.4E-02	6.3E-03	3.5E-02	3.1E-03	2.7E-02	1.7E-02	3.1E-02	9.3E-03	3.2E-02	1.6E-02
Guanidinosuccinic acid	7.2E-04	4.1E-04	1.2E-03	2.6E-04	3.7E-04	4.8E-04	1.4E-04	1.2E-04	9.4E-04	N.A.	4.4E-04	1.0E-04
Guanine	1.8E-03	4.1E-04	1.5E-03	5.1E-04	2.8E-03	1.4E-03	2.7E-03	8.0E-04	4.6E-03	2.4E-04	2.5E-03	1.9E-03
Guanosine	1.6E-03	3.3E-04	1.9E-03	2.5E-04	2.2E-03	1.7E-04	2.3E-03	5.9E-04	2.3E-03	3.3E-04	2.6E-03	1.5E-03
Hexanoic acid	1.2E-04	N.A.	1.1E-04	9.8E-06	1.2E-04	2.8E-05	1.3E-04	2.8E-05	1.4E-04	8.2E-06	1.3E-04	2.4E-05
His	2.7E-02	5.0E-03	2.6E-02	5.2E-03	7.0E-02	2.6E-02	4.4E-02	2.3E-02	6.7E-02	1.9E-02	4.9E-02	6.5E-03
Histamine	1.4E-03	1.1E-03	4.6E-04	8.3E-05	9.1E-03	1.5E-02	8.8E-04	5.4E-04	1.1E-03	1.6E-03	2.9E-04	1.4E-04
Homoserine	N.D.	N.A.	2.4E-04	8.5E-05	N.D.	N.A.	N.D.	N.A.	N.D.	N.A.	N.D.	N.A.
Hydroxyproline	9.3E-04	4.2E-04	1.9E-03	6.1E-04	1.7E-03	8.0E-04	8.3E-03	5.5E-03	1.6E-03	4.3E-04	9.6E-03	1.3E-02

Hypotaurine	8.7E-01	2.2E-01	8.2E-01	3.4E-01	3.8E-01	4.9E-02	5.0E-01	9.4E-02	5.0E-01	9.7E-02	5.7E-01	3.1E-01
Hypoxanthine	4.0E-03	7.7E-04	2.7E-03	1.4E-03	5.6E-03	5.4E-04	4.6E-03	1.3E-04	5.2E-03	1.8E-03	8.0E-03	4.5E-03
IDP	1.1E-03	5.5E-04	1.2E-03	1.4E-04	1.5E-03	9.3E-04	3.9E-04	3.1E-04	1.5E-03	8.5E-04	5.0E-04	3.3E-04
IMP	1.6E-02	2.7E-03	3.1E-02	1.2E-02	3.4E-02	1.2E-02	1.2E-02	1.0E-02	3.5E-02	8.6E-03	3.1E-02	3.4E-02
Ile	8.0E-02	8.6E-03	9.2E-02	2.7E-02	9.1E-02	1.4E-02	9.2E-02	4.0E-02	9.4E-02	2.0E-02	1.1E-01	2.5E-02
Imidazole-4-acetic acid	2.8E-04	5.9E-05	2.9E-04	1.4E-05	4.6E-04	2.6E-04	N.D.	N.A.	N.D.	N.A.	N.D.	N.A.
Inosine	1.5E-02	2.3E-03	1.1E-02	1.5E-03	1.3E-02	1.4E-03	1.1E-02	1.7E-03	1.3E-02	2.4E-03	1.4E-02	8.8E-03
Isethionic acid	1.0E-04	4.9E-06	1.8E-04	1.0E-04	8.9E-05	5.3E-05	1.2E-04	N.A.	1.1E-04	2.4E-05	2.3E-04	3.4E-05
Isoamylamine	N.D.	N.A.	N.D.	N.A.	1.1E-03	N.A.	N.D.	N.A.	2.1E-03	1.7E-03	1.6E-03	1.6E-04
Isobutyrylcarnitine	3.2E-04	1.3E-04	5.0E-04	2.1E-04	7.5E-04	3.2E-04	4.9E-04	8.0E-05	2.1E-04	N.A.	3.9E-04	1.4E-04
Isoglutamic acid	6.3E-04	N.A.	2.7E-03	3.4E-03	N.D.	N.A.	N.D.	N.A.	N.D.	N.A.	N.D.	N.A.
Isovalerylcarnitine	2.6E-04	9.8E-05	2.9E-04	1.4E-04	2.7E-04	1.2E-04	4.8E-04	4.0E-04	2.3E-04	8.5E-05	2.1E-04	3.5E-05
Lactic acid	2.0E-03	3.4E-04	2.2E-03	6.6E-04	2.0E-03	1.7E-04	3.2E-03	3.4E-04	2.1E-03	4.4E-04	2.7E-03	8.8E-04
Lauric acid	3.3E-04	2.2E-05	3.1E-04	2.2E-05	2.9E-04	3.3E-05	3.4E-04	1.5E-05	2.7E-04	3.0E-05	2.6E-04	4.3E-05
Leu	1.4E-01	2.4E-03	1.5E-01	3.4E-02	1.6E-01	1.1E-02	1.5E-01	5.5E-02	1.7E-01	3.7E-02	1.8E-01	5.0E-02
Lys	1.5E-01	1.6E-02	1.9E-01	8.5E-02	2.2E-01	1.2E-01	1.4E-01	2.1E-02	1.7E-01	3.8E-02	1.8E-01	4.3E-02
Malic acid	5.1E-02	1.8E-02	4.9E-02	1.3E-02	9.1E-02	2.2E-02	8.0E-02	9.7E-03	7.9E-02	7.6E-03	1.1E-01	6.6E-02
Met	3.7E-02	8.4E-03	4.5E-02	1.5E-02	2.5E-02	2.3E-03	2.4E-02	1.1E-02	3.0E-02	9.4E-03	3.1E-02	1.3E-02
Methanesulfonic acid	1.7E-03	5.8E-04	N.D.	N.A.	N.D.	N.A.	N.D.	N.A.	N.D.	N.A.	N.D.	N.A.
Methionine sulfoxide	6.2E-04	1.8E-04	5.8E-04	1.9E-04	1.9E-03	5.3E-04	1.0E-03	1.9E-04	1.3E-03	2.2E-04	1.2E-03	1.6E-04
Mevalolactone	4.2E-03	N.A.	3.2E-03	N.A.	2.7E-02	1.3E-02	1.6E-02	7.8E-03	2.9E-02	7.7E-03	2.0E-02	8.4E-03
N,*N*-Dimethylglycine	6.2E-03	5.4E-03	6.9E-03	3.1E-03	2.2E-03	1.4E-03	4.8E-03	4.9E-04	1.2E-03	2.3E-04	7.9E-03	7.6E-03
N-Acetylalanine	6.0E-05	1.4E-05	5.7E-05	9.7E-06	7.1E-05	1.6E-05	8.7E-05	5.1E-05	8.6E-05	3.1E-05	8.7E-05	1.7E-05
N-Acetylaspartic acid	3.4E-05	1.0E-05	1.3E-04	1.1E-04	5.8E-05	2.5E-05	1.1E-04	1.2E-04	5.8E-05	7.6E-07	9.5E-05	6.6E-05
N-Acetylglucosamine 1-phosphate	4.2E-04	9.1E-05	3.4E-04	4.8E-05	4.7E-04	1.3E-04	4.3E-04	6.7E-05	5.0E-04	1.3E-04	4.6E-04	8.5E-05
N-Acetylglucosamine 6-phosphate	1.7E-04	3.7E-05	2.6E-04	3.4E-05	2.6E-04	1.9E-05	2.1E-04	7.8E-05	3.3E-04	6.0E-05	3.4E-04	6.6E-05
N-Acetylglutamic acid	7.5E-05	7.3E-05	1.1E-04	6.6E-06	8.8E-05	4.7E-05	1.4E-04	1.1E-04	1.2E-04	4.3E-05	9.7E-05	4.2E-06
N-Acetylglycine	3.5E-05	N.A.	9.1E-05	2.3E-05	9.6E-05	4.4E-05	4.5E-05	1.6E-05	4.3E-05	2.2E-05	5.0E-05	N.A.

N-Acetyllysine	1.1E-03	3.4E-04	1.0E-03	7.3E-05	8.2E-04	3.4E-04	5.3E-04	2.2E-04	9.8E-04	2.9E-04	6.7E-04	6.0E-05
N-Methylalanine	1.2E-03	7.0E-04	1.3E-03	1.2E-04	N.D.	N.A.	6.7E-04	N.A.	N.D.	N.A.	3.2E-04	N.A.
N-Methylaspartic acid	1.1E-03	5.6E-04	1.2E-03	5.2E-04	N.D.	N.A.	N.D.	N.A.	5.2E-04	2.4E-04	N.D.	N.A.
N-Methylproline	1.1E-02	9.1E-03	1.2E-02	3.4E-03	2.6E-03	5.8E-04	5.8E-03	3.9E-03	5.1E-03	3.0E-03	3.7E-03	1.7E-03
N^8-Acetylspermidine	8.8E-05	N.A.	2.3E-04	2.3E-04	1.0E-04	N.A.	2.3E-04	1.3E-04	1.1E-04	3.2E-05	1.6E-04	4.2E-05
NAD^+	3.9E-03	4.9E-04	3.3E-03	4.3E-04	3.7E-03	5.8E-04	3.4E-03	2.1E-04	3.5E-03	3.9E-04	3.0E-03	1.3E-03
$NADP^+$	3.4E-04	8.4E-05	3.7E-04	9.6E-05	3.9E-04	7.6E-05	3.9E-04	5.6E-05	3.9E-04	1.1E-04	3.1E-04	6.0E-05
Nicotinamide	5.6E-04	4.6E-05	5.3E-04	1.2E-04	5.5E-04	2.7E-04	9.1E-04	4.0E-04	6.4E-04	3.2E-04	5.9E-04	3.7E-04
Nicotinic acid	6.3E-03	6.2E-04	5.9E-03	1.1E-03	8.1E-03	2.2E-03	9.2E-03	3.7E-03	1.1E-02	9.2E-04	1.3E-02	2.7E-03
Norspermidine	1.5E-04	1.2E-05	1.7E-04	8.4E-05	3.3E-04	1.3E-04	3.4E-04	5.2E-05	2.2E-04	4.0E-05	2.9E-04	2.0E-04
N_{ω}-Methylarginine	2.3E-04	4.0E-05	2.4E-04	2.6E-05	2.8E-04	2.8E-05	3.2E-04	2.0E-04	2.4E-04	3.8E-05	2.8E-04	1.5E-04
O-Acetylcarnitine	1.2E-02	2.8E-03	1.4E-02	4.1E-03	2.0E-02	2.1E-03	1.9E-02	6.1E-03	1.8E-02	2.4E-03	2.2E-02	6.5E-03
Octopamine	8.3E-04	2.8E-04	9.6E-04	2.0E-04	6.0E-04	2.0E-05	1.1E-03	8.5E-06	6.0E-04	7.7E-05	1.0E-03	1.1E-04
Octopine	1.8E-02	1.1E-02	9.6E-03	1.4E-03	1.9E-02	6.0E-03	1.3E-02	4.4E-03	1.6E-02	8.9E-03	2.9E-02	6.3E-03
Ophthalmic acid	9.5E-04	1.8E-04	3.9E-04	2.0E-04	5.7E-03	1.8E-03	4.2E-03	4.2E-03	3.8E-03	1.9E-03	6.4E-03	4.5E-03
Ornithine	1.7E-02	4.3E-03	2.2E-02	1.1E-02	2.1E-02	1.4E-02	1.2E-02	3.0E-03	1.9E-02	8.8E-03	1.6E-02	6.6E-03
PRPP	1.8E-04	8.9E-05	1.3E-04	2.8E-05	1.9E-04	3.1E-05	1.5E-04	4.0E-05	1.3E-04	2.9E-05	1.1E-04	1.1E-05
Pantothenic acid	5.0E-04	2.0E-04	4.2E-04	1.2E-04	9.7E-04	2.9E-04	6.5E-04	1.9E-04	9.1E-04	8.6E-05	4.7E-04	6.1E-05
Pelargonic acid	1.8E-04	4.2E-05	2.3E-04	3.9E-05	2.1E-04	N.A.	2.1E-04	1.9E-05	1.7E-04	N.A.	1.9E-04	2.2E-05
Phe	4.3E-02	3.8E-03	4.8E-02	2.6E-03	6.8E-02	2.6E-03	5.5E-02	1.8E-02	7.1E-02	2.0E-02	7.3E-02	2.5E-02
Phosphoenolpyruvic acid	1.1E-03	3.7E-04	1.1E-03	3.5E-04	8.0E-04	1.2E-04	7.6E-04	1.5E-04	1.1E-03	7.1E-05	1.2E-03	8.1E-05
Phosphorylcholine	2.1E-02	3.5E-03	2.5E-02	1.6E-03	2.1E-02	1.3E-02	1.7E-02	3.8E-03	1.7E-02	8.9E-03	1.5E-02	2.2E-03
Pipecolic acid	5.7E-04	1.1E-04	5.5E-03	8.1E-03	7.3E-04	1.5E-04	9.8E-04	6.1E-04	1.2E-03	7.9E-04	2.2E-03	2.1E-03
Pro	5.3E-02	9.8E-03	5.4E-02	1.8E-02	1.6E-01	2.1E-02	1.6E-01	8.2E-02	1.2E-01	1.3E-02	1.3E-01	4.6E-02
Prostaglandin E_2	1.6E-04	8.3E-05	1.6E-04	1.4E-05	4.4E-04	8.1E-05	5.0E-04	4.6E-04	5.1E-04	1.8E-04	6.5E-04	5.3E-04
Prostaglandin $F_{2\alpha}$	1.2E-04	3.9E-05	1.2E-04	1.7E-05	2.8E-04	6.8E-06	2.6E-04	1.9E-04	3.6E-04	8.8E-05	4.2E-04	3.1E-04
Putrescine	3.3E-04	4.3E-05	5.1E-04	2.1E-04	6.5E-04	1.2E-04	8.1E-04	2.4E-04	5.9E-04	9.9E-05	5.6E-04	1.0E-04
Pyruvic acid	1.7E-03	5.2E-04	1.6E-03	5.1E-04	2.4E-03	8.5E-05	2.4E-03	1.4E-03	1.9E-03	1.1E-04	2.1E-03	3.2E-04

Ribose 5-phosphate	6.5E-04	3.1E-05	7.1E-04	2.4E-04	1.0E-03	1.3E-04	8.6E-04	2.7E-04	7.4E-04	1.3E-04	1.1E-03	5.1E-04
Ribulose 1,5-diphosphate	7.0E-05	5.7E-06	6.3E-05	4.9E-06	7.6E-05	4.6E-06	7.9E-05	2.9E-05	8.3E-05	1.0E-05	9.7E-05	6.6E-06
Ribulose 5-phosphate	1.5E-03	1.7E-04	1.3E-03	3.4E-04	2.1E-03	3.3E-04	1.7E-03	6.6E-04	1.9E-03	4.8E-04	3.0E-03	2.4E-03
S-Adenosylmethionine	1.7E-04	1.3E-05	1.7E-04	7.0E-05	2.1E-04	1.5E-05	2.4E-04	1.5E-04	2.0E-04	3.7E-06	1.7E-04	4.7E-05
Saccharopine	6.2E-04	2.8E-04	6.1E-04	1.9E-05	4.8E-04	8.7E-05	5.3E-04	3.6E-06	3.5E-04	9.2E-05	5.3E-04	1.6E-04
Sarcosine	5.9E-03	9.3E-04	2.5E-02	2.8E-02	N.D.	N.A.	N.D.	N.A.	2.2E-03	N.A.	3.7E-03	2.8E-03
Sedoheptulose 7-phosphate	8.5E-04	2.8E-04	8.4E-04	3.1E-04	1.9E-03	1.2E-04	1.3E-03	1.8E-04	1.6E-03	3.3E-04	1.8E-03	5.5E-04
Ser	4.7E-02	7.8E-03	4.4E-02	1.3E-02	8.3E-02	2.0E-02	4.6E-02	4.3E-03	8.1E-02	5.5E-03	5.0E-02	1.3E-02
Spermidine	1.8E-03	2.5E-04	1.7E-03	3.6E-04	3.4E-03	1.4E-03	2.7E-03	2.9E-04	2.7E-03	4.1E-04	2.8E-03	1.1E-03
Spermine	6.6E-04	2.7E-04	6.9E-04	1.5E-04	8.1E-04	3.8E-04	5.9E-04	2.1E-04	6.4E-04	1.4E-04	6.3E-04	8.5E-05
Stachydrine	6.8E-01	8.7E-02	7.2E-01	1.3E-01	4.2E-01	1.0E-01	5.1E-01	2.7E-02	5.4E-01	8.4E-02	4.5E-01	1.0E-01
Succinic acid	6.2E-02	1.5E-02	7.1E-02	1.5E-02	7.3E-02	7.8E-03	1.1E-01	6.7E-02	9.9E-02	3.4E-02	1.1E-01	1.8E-02
Tartaric acid	N.D.	N.A.	N.D.	N.A.	1.0E-04	N.A.	1.5E-03	N.A.	N.D.	N.A.	4.6E-04	N.A.
Taurine	7.5E-01	1.4E-02	7.6E-01	4.5E-02	6.6E-01	4.0E-02	7.6E-01	2.8E-02	6.5E-01	8.6E-03	6.9E-01	2.1E-02
Thiaproline	7.7E-04	6.3E-05	5.5E-04	2.0E-04	4.5E-04	3.8E-05	6.3E-04	1.6E-04	6.7E-04	2.0E-04	7.3E-04	2.2E-04
Thr	4.7E-02	5.0E-03	5.1E-02	1.0E-02	7.4E-02	1.4E-02	6.1E-02	8.1E-03	7.2E-02	1.7E-02	6.4E-02	1.3E-02
Trigonelline	1.2E-02	7.7E-03	1.5E-02	1.1E-03	4.4E-02	8.7E-03	2.1E-02	4.6E-03	4.1E-02	1.5E-02	4.0E-02	1.1E-02
Trimethylamine *N*-oxide	7.3E-03	5.8E-03	9.3E-03	2.9E-03	3.2E-03	1.9E-03	4.9E-03	2.2E-03	2.2E-03	7.7E-04	5.0E-03	3.6E-03
Trp	4.9E-03	7.5E-04	5.4E-03	5.2E-04	7.7E-03	5.6E-04	7.5E-03	2.6E-03	8.3E-03	2.1E-03	8.4E-03	1.9E-03
Tyr	8.2E-03	6.1E-04	9.9E-03	2.7E-03	1.1E-02	2.2E-03	9.5E-03	3.8E-03	1.3E-02	3.0E-03	1.3E-02	2.7E-03
UDP	2.2E-03	2.4E-04	1.8E-03	6.3E-04	2.3E-03	1.0E-03	1.8E-03	9.6E-04	1.9E-03	4.9E-04	1.7E-03	1.3E-03
UDP-*N*-acetylgalactosamine UDP-*N*-acetylglucosamine	1.9E-02	3.6E-03	2.0E-02	8.3E-04	2.7E-02	5.2E-03	2.7E-02	6.2E-03	2.4E-02	7.1E-03	2.7E-02	8.9E-04
UDP-glucuronic acid	3.9E-04	1.0E-04	5.1E-04	1.3E-04	6.4E-04	2.7E-04	5.4E-04	9.7E-05	5.0E-04	9.1E-05	5.8E-04	9.0E-05
UMP	2.4E-03	5.8E-04	3.7E-03	6.6E-04	5.5E-03	2.4E-03	3.7E-03	8.7E-04	4.5E-03	8.5E-04	4.1E-03	1.3E-03
UTP	1.6E-03	9.2E-04	7.5E-04	5.1E-04	2.0E-03	2.0E-03	1.0E-03	1.3E-03	9.2E-04	6.5E-04	9.7E-04	2.3E-04

Uric acid	1.8E-03	1.4E-03	1.3E-03	3.2E-04	4.4E-04	2.9E-04	1.1E-03	1.1E-03	5.4E-04	1.8E-04	6.9E-04	2.1E-04
Uridine	8.7E-04	6.7E-05	9.8E-04	1.6E-04	1.3E-03	1.9E-04	1.5E-03	1.3E-04	1.7E-03	2.7E-05	1.4E-03	5.9E-04
Val	8.6E-02	3.7E-03	9.8E-02	2.6E-02	1.1E-01	1.7E-02	1.0E-01	3.0E-02	1.1E-01	1.8E-02	1.2E-01	2.3E-02
$C_4H_9NO_4S$	1.5E-04	9.2E-06	1.6E-04	4.2E-05	2.6E-04	1.9E-04	2.3E-04	1.3E-04	1.5E-04	7.7E-06	1.4E-04	5.3E-05
$C_6H_8O_7$	1.0E-03	1.4E-04	8.6E-04	6.3E-05	2.2E-03	5.9E-04	2.2E-03	2.1E-04	1.8E-03	2.7E-04	1.7E-03	3.2E-04
$C_9H_{13}N_3O_5$	3.6E-03	6.2E-04	4.0E-03	2.5E-04	3.9E-03	2.1E-03	3.2E-03	7.6E-04	2.8E-03	1.3E-03	2.7E-03	2.5E-04
$C_7H_{16}O_{13}P_2$	1.0E-04	5.5E-05	8.9E-05	N.A.	2.8E-04	1.0E-04	2.0E-04	3.0E-05	2.7E-04	4.7E-05	3.5E-04	7.4E-05
$C_{11}H_{20}N_4O_{11}P_2$	1.0E-03	2.3E-04	1.2E-03	6.0E-05	1.2E-03	1.3E-04	1.1E-03	2.5E-04	1.7E-03	1.9E-04	1.1E-03	3.6E-04
$C_5H_8N_2O_2$	9.6E-04	8.9E-05	2.1E-03	2.3E-03	2.0E-03	9.0E-04	2.6E-03	1.3E-03	2.9E-03	1.3E-03	1.4E-03	5.2E-04
$C_{10}H_{19}NO_4$	1.0E-02	2.3E-03	8.5E-03	1.4E-03	6.6E-03	1.4E-03	7.6E-03	1.6E-03	7.2E-03	3.8E-03	9.5E-03	3.8E-03
$C_{12}H_{24}N_2O_8$	2.0E-04	6.8E-06	2.4E-04	5.5E-05	3.1E-04	7.0E-05	3.9E-04	1.4E-04	3.2E-04	6.4E-05	4.2E-04	2.4E-04
Xanthine	9.6E-04	2.3E-04	8.5E-04	2.5E-04	1.1E-03	2.4E-04	1.0E-03	2.2E-04	1.2E-03	3.0E-04	1.5E-03	3.9E-04
allo-Threonine	3.2E-04	5.1E-05	3.5E-04	5.9E-05	3.0E-02	5.2E-02	4.3E-04	7.4E-05	4.6E-02	6.4E-02	3.2E-04	8.8E-05
dTDP	N.D.	N.A.	N.D.	N.A.	N.D.	N.A.	N.D.	N.A.	7.0E-05	N.A.	N.D.	N.A.
dTMP	3.4E-05	9.5E-06	4.2E-05	2.1E-05	6.0E-05	1.2E-05	5.7E-05	2.5E-05	9.3E-05	4.5E-05	7.0E-05	2.6E-05
myo-Inositol 1-phosphate *myo*-Inositol 3-phosphate	5.2E-04	1.7E-04	5.0E-04	8.0E-05	9.0E-04	9.3E-05	7.2E-04	9.5E-05	1.1E-03	2.4E-04	9.6E-04	1.9E-04
myo-Inositol 2-phosphate	3.5E-04	8.7E-05	3.4E-04	1.7E-04	4.5E-04	1.1E-04	4.6E-04	1.5E-05	4.8E-04	1.6E-04	5.8E-04	2.7E-04
threo-β-Methylaspartic acid	1.9E-03	4.3E-05	2.2E-03	2.4E-04	8.6E-04	2.2E-04	1.3E-03	3.2E-04	8.6E-04	3.0E-04	1.2E-03	2.4E-04
β-Ala	6.3E-02	1.7E-02	5.5E-02	2.0E-02	1.8E-01	1.1E-01	1.1E-01	5.9E-03	1.1E-01	8.6E-02	1.3E-01	6.5E-02
β-Tyr	4.4E-04	4.0E-05	6.4E-04	3.3E-04	1.8E-04	N.A.	3.8E-04	3.9E-04	1.2E-04	3.2E-05	1.8E-04	5.4E-05
γ-Butyrobetaine	8.5E-02	1.4E-02	8.0E-02	1.2E-02	4.8E-02	9.8E-03	6.9E-02	7.0E-03	4.4E-02	9.1E-03	7.3E-02	2.4E-02
γ-Glu-2-aminobutyric acid	3.9E-03	6.2E-04	2.6E-03	5.0E-04	1.2E-02	2.4E-03	4.7E-03	1.2E-03	7.9E-03	2.6E-03	9.8E-03	5.1E-03
γ-Glu-Cys	9.5E-04	N.A.	N.D.	N.A.	9.4E-03	7.0E-03	1.4E-02	1.3E-02	7.7E-03	5.4E-03	3.7E-03	1.5E-03
γ-Glu-Val-Gly	N.D.	N.A.	N.D.	N.A.	4.8E-04	2.9E-04	2.8E-04	3.3E-05	2.8E-04	6.5E-05	2.5E-04	1.8E-04

N.D.: Not detected
N.A.: Not available

Appendix S2 Concentrations of major metabolites in the largest and smallest clams of each feeding group.

Metabolite	Concentration (nmol/g)											
	Unfed (largest)		Unfed (smallest)		*C. neogracile* (largest)		*C. neogracile* (smallest)		*C. neogracile* + AHs (largest)		*C. neogracile* + AHs (smallest)	
	Mean	S.D.	Mean	S.D.	Mean	S.D.	Mean	S.D.	Mean	S.D.	Mean	S.D.
2-Hydroxybutyric acid	N.D.	N.A.	N.D.	N.A.	N.D.	N.A.	N.D.	N.A.	N.D.	N.A.	N.D.	N.A.
2-Oxoglutaric acid	N.D.	N.A.	N.D.	N.A.	N.D.	N.A.	N.D.	N.A.	N.D.	N.A.	N.D.	N.A.
2-Oxoisovaleric acid	N.D.	N.A.	N.D.	N.A.	N.D.	N.A.	N.D.	N.A.	N.D.	N.A.	N.D.	N.A.
2-Phosphoglyceric acid	15.0	5.4	15.0	4.8	13.0	1.4	12.0	2.1	14.0	0.5	16.0	0.4
3-Hydroxybutyric acid	N.D.	N.A.	N.D.	N.A.	N.D.	N.A.	N.D.	N.A.	N.D.	N.A.	N.D.	N.A.
3-Phosphoglyceric acid	164.0	65.0	172.0	58.0	136.0	14.0	127.0	22.0	156.0	3.7	182.0	6.0
6-Phosphogluconic acid	108.0	37.0	123.0	17.0	191.0	26.0	154.0	44.0	230.0	67.0	234.0	85.0
ADP	923.0	140.0	827.0	146.0	997.0	367.0	1,133.0	100.0	1,085.0	95.0	1,031.0	662.0
AMP	248.0	217.0	374.0	200.0	455.0	280.0	746.0	528.0	669.0	246.0	671.0	156.0
ATP	952.0	381.0	512.0	343.0	843.0	357.0	714.0	629.0	574.0	173.0	511.0	425.0
Acetyl CoA_divalent	0.5	0.02	N.D.	N.A.	0.7	N.A.	0.6	N.A.	0.4	0.2	0.3	0.05
Adenine	N.D.	N.A.	0.4	N.A.	0.5	N.A.	N.D.	N.A.	0.7	0.2	0.7	N.A.
Adenosine	11.0	5.8	17.0	7.0	19.0	3.5	25.0	14.0	15.0	5.2	19.0	5.0
Ala	6,094.0	1,021.0	5,691.0	1,373.0	11,154.0	1,183.0	9,308.0	2,221.0	9,805.0	1,114.0	8,476.0	1,013.0
Anthranilic acid	N.D.	N.A.	N.D.	N.A.	235.0	N.A.	N.D.	N.A.	247.0	N.A.	N.D.	N.A.

Arg	5,515.0	543.0	4,897.0	1,259.0	5,384.0	1,051.0	4,283.0	338.0	5,835.0	327.0	5,265.0	457.0
Asn	191.0	71.0	208.0	114.0	332.0	183.0	128.0	110.0	659.0	428.0	162.0	263.0
Asp	6,428.0	475.0	5,768.0	834.0	8,343.0	1,194.0	8,310.0	2,236.0	7,614.0	1,580.0	6,418.0	1,602.0
Betaine aldehyde + H_2O	N.D.	N.A.	N.D.	N.A.	N.D.	N.A.	N.D.	N.A.	N.D.	N.A.	N.D.	N.A.
Betaine	43,507.0	4,549.0	44,785.0	2,872.0	32,260.0	2,886.0	38,878.0	4,103.0	34,880.0	1,423.0	37,412.0	4,266.0
CDP	4.4	1.0	2.5	1.0	11.0	5.8	5.4	2.2	5.9	1.6	3.7	2.7
CMP	5.0	0.9	5.4	2.0	16.0	12.0	8.6	2.7	11.0	4.5	7.5	0.4
CTP	3.2	1.5	1.5	1.0	9.8	10.0	3.9	3.9	2.8	1.8	2.3	0.9
Carnosine	N.D.	N.A.	N.D.	N.A.	N.D.	N.A.	N.D.	N.A.	N.D.	N.A.	N.D.	N.A.
Choline	58.0	6.9	62.0	13.0	69.0	12.0	79.0	9.7	62.0	16.0	66.0	3.4
Citric acid	61.0	7.0	65.0	29.0	100.0	28.0	102.0	13.0	77.0	12.0	92.0	15.0
Citrulline	6.8	1.1	7.0	0.9	12.0	2.7	11.0	6.1	12.0	2.1	11.0	3.2
CoA divalent	N.D.	N.A.	N.D.	N.A.	1.7	1.5	1.8	N.A.	3.2	N.A.	1.3	0.7
Creatine	24.0	39.0	18.0	N.A.	4.8	3.7	1.1	0.04	13.0	18.0	1.6	N.A.
Creatinine	1.4	N.A.	N.D.	N.A.	N.D.	N.A.	N.D.	N.A.	N.D.	N.A.	N.D.	N.A.
Cys	N.D.	N.A.	N.D.	N.A.	N.D.	N.A.	N.D.	N.A.	N.D.	N.A.	N.D.	N.A.
Cytidine	N.D.	N.A.	N.D.	N.A.	14	N.A.	N.D.	N.A.	N.D.	N.A.	N.D.	N.A.
Cytosine	1.7	N.A.	3.1	N.A.	N.D.	N.A.	N.D.	N.A.	N.D.	N.A.	N.D.	N.A.
Dihydroxyacetone phosphate	50.0	19.0	52.0	24.0	136.0	40.0	111.0	21.0	120.0	19.0	126.0	17.0
Erythrose 4-phosphate	N.D.	N.A.	N.D.	N.A.	N.D.	N.A.	N.D.	N.A.	N.D.	N.A.	N.D.	N.A.
Fructose 1,6-diphosphate	4.6	1.8	4.4	1.3	21.0	9.4	11.0	4.6	22.0	9.6	22.0	5.2
Fructose 6-phosphate	97.0	48.0	78.0	18.0	175.0	15.0	112.0	20.0	151.0	25.0	158.0	93.0
Fumaric acid	143.0	67.0	129.0	29.0	240.0	44.0	220.0	58.0	229.0	13.0	305.0	181.0
GABA	3.9	0.6	5.1	0.6	5.8	0.7	6.2	2.0	5.2	0.2	8.0	1.4
GDP	52.0	1.6	50.0	12.0	43.0	7.6	45.0	16.0	50.0	5.6	42.0	23.0

GMP	100.0	31.0	156.0	52.0	178.0	16.0	175.0	84.0	184.0	25.0	174.0	18.0
GTP	51.0	20.0	27.0	24.0	47.0	32.0	26.0	26.0	26.0	13.0	26.0	5.2
Gln	1,128.0	606.0	968.0	402.0	5,211.0	2,472.0	3,173.0	2,645.0	6,054.0	1,098.0	4,419.0	5,311.0
Glu	7,347.0	510.0	7,258.0	1,204.0	9,017.0	470.0	8,838.0	502.0	10,168.0	1,359.0	8,934.0	1,774.0
Gluconic acid	12.0	0.9	11.0	1.5	16.0	4.5	19.0	7.0	18.0	2.2	19.0	4.8
Glucose 1-phosphate	21.0	12.0	17.0	5.9	46.0	5.7	27.0	0.4	46.0	19.0	54.0	52.0
Glucose 6-phosphate	385.0	223.0	298.0	103.0	653.0	92.0	410.0	58.0	565.0	118.0	587.0	377.0
Glutathione (GSH)	N.D.	N.A.	N.D.	N.A.	31.0	27.0	31.0	11.0	30.0	37.0	25.0	23.0
Glutathione (GSSG)_divalent	19.0	11.0	8.5	6.0	38.0	27.0	41.0	41.0	43.0	11.0	47.0	28.0
Gly	13,362.0	3,496.0	9,861.0	4,206.0	33,081.0	5,070.0	23,202.0	10,418.0	38,840.0	1,668.0	28,633.0	7,007.0
Glyceraldehyde 3-phosphate	N.D.	N.A.	N.D.	N.A.	N.D.	N.A.	N.D.	N.A.	N.D.	N.A.	N.D.	N.A.
Glycerol 3-phosphate	74.0	9.3	58.0	18.0	95.0	15.0	81.0	3.6	92.0	12.0	93.0	45.0
Glycolic acid	N.D.	N.A.	N.D.	N.A.	N.D.	N.A.	N.D.	N.A.	N.D.	N.A.	N.D.	N.A.
Glyoxylic acid	N.D.	N.A.	N.D.	N.A.	N.D.	N.A.	N.D.	N.A.	N.D.	N.A.	N.D.	N.A.
Guanine	14.0	3.3	12.0	4.2	23.0	11.0	22.0	6.5	37.0	1.9	20.0	15.0
Guanosine	15.0	3.0	17.0	2.3	20.0	1.6	21.0	5.4	21.0	3.1	24.0	14.0
His	188.0	35.0	184.0	37.0	494.0	186.0	311.0	163.0	477.0	137.0	350.0	46.0
Homoserine	N.D.	N.A.	1.6	0.6	N.D.	N.A.	N.D.	N.A.	N.D.	N.A.	N.D.	N.A.
Hydroxyproline	5.8	2.6	12.0	3.8	10.0	5.0	52.0	34.0	10.0	2.7	60.0	81.0
Hypoxanthine	33.0	6.3	22.0	11.0	45.0	4.4	38.0	1.0	42.0	15.0	66.0	37.0
IMP	458.0	76.0	876.0	332.0	968.0	344.0	334.0	281.0	986.0	243.0	861.0	946.0
Ile	208.0	22.0	239.0	72.0	236.0	36.0	239.0	104.0	246.0	52.0	285.0	66.0
Inosine	172.0	27.0	129.0	17.0	150.0	16.0	130.0	19.0	155.0	28.0	162.0	102.0
Isocitric acid	N.D.	N.A.	N.D.	N.A.	N.D.	N.A.	N.D.	N.A.	N.D.	N.A.	N.D.	N.A.
Lactic acid	72.0	12.0	78.0	23.0	69.0	5.9	113.0	12.0	74.0	15.0	97.0	31.0

Leu	309.0	5.4	338.0	77.0	358.0	25.0	340.0	124.0	387.0	83.0	411.0	113.0
Lys	1,104.0	119.0	1,393.0	625.0	1,637.0	908.0	1,059.0	156.0	1,228.0	276.0	1,342.0	315.0
Malic acid	929.0	335.0	894.0	230.0	1,665.0	405.0	1,455.0	177.0	1,450.0	140.0	2,012.0	1,205.0
Malonyl CoA_divalent	N.D.	N.A.	N.D.	N.A.	N.D.	N.A.	N.D.	N.A.	N.D.	N.A.	N.D.	N.A.
Met	218.0	49.0	265.0	88.0	149.0	13.0	140.0	67.0	175.0	55.0	182.0	74.0
N,*N*-Dimethylglycine	31.0	27.0	35.0	16.0	11.0	6.8	24.0	2.4	6.0	1.1	39.0	38.0
NAD^+	125.0	16.0	108.0	14.0	120.0	19.0	110.0	6.6	114.0	13.0	97.0	42.0
$NADP^+$	9.8	2.4	10.0	2.7	11.0	2.2	11.0	1.6	11.0	3.1	8.9	1.7
Ornithine	107.0	27.0	136.0	67.0	132.0	88.0	77.0	19.0	117.0	54.0	101.0	41.0
PRPP	5.3	2.7	3.8	0.8	5.6	0.9	4.4	1.2	3.8	0.9	3.4	0.3
Phe	156.0	14.0	175.0	9.3	245.0	9.6	198.0	66.0	259.0	73.0	265.0	92.0
Phosphoenolpyruvic acid	38.0	12.0	39.0	12.0	27.0	4.0	26.0	5.1	37.0	2.4	42.0	2.8
Pro	207.0	38.0	212.0	72.0	624.0	83.0	625.0	323.0	483.0	53.0	522.0	181.0
Putrescine	3.2	0.4	4.9	2.0	6.3	1.2	7.8	2.3	5.7	1.0	5.4	1.0
Pyruvic acid	113.0	35.0	108.0	35.0	161.0	5.8	167.0	93.0	128.0	7.3	140.0	22.0
Ribose 5-phosphate	18.0	0.8	19.0	6.6	27.0	3.6	23.0	7.2	20.0	3.6	30.0	14.0
Ribulose 5-phosphate	48.0	5.5	42.0	11.0	68.0	10.0	54.0	21.0	60.0	15.0	96.0	77.0
S-Adenosylmethionine	4.5	0.4	4.7	1.9	5.6	0.4	6.4	4.0	5.3	0.10	4.5	1.3
Sarcosine	31.0	4.9	132.0	147.0	N.D.	N.A.	N.D.	N.A.	12.0	N.A.	19.0	15.0
Sedoheptulose 7-phosphate	23.0	7.6	22.0	8.4	50.0	3.3	35.0	4.7	43.0	8.7	48.0	15.0
Ser	461.0	76.0	432.0	127.0	809.0	197.0	449.0	42.0	793.0	53.0	490.0	122.0
Spermidine	11.0	1.4	10.0	2.1	20.0	8.5	16.0	1.7	16.0	2.4	17.0	6.5
Spermine	21.0	8.4	22.0	4.8	25.0	12.0	19.0	6.7	20.0	4.2	20.0	2.7
Succinic acid	1,712.0	419.0	1,958.0	404.0	2,006.0	216.0	3,106.0	1,851.0	2,733.0	951.0	3,133.0	485.0

Thr	313.0	33.0	337.0	68.0	486.0	92.0	405.0	54.0	473.0	110.0	426.0	84.0
Thymidine	N.D.	N.A.	N.D.	N.A.	N.D.	N.A.	N.D.	N.A.	N.D.	N.A.	N.D.	N.A.
Thymine	N.D.	N.A.	N.D.	N.A.	N.D.	N.A.	N.D.	N.A.	N.D.	N.A.	N.D.	N.A.
Trp	25.0	3.9	28.0	2.7	40.0	2.9	39.0	14.0	43.0	11.0	44.0	9.9
Tyr	53.0	3.9	63.0	17.0	73.0	14.0	61.0	25.0	83.0	19.0	83.0	18.0
Tyramine	N.D.	N.A.	N.D.	N.A.	N.D.	N.A.	N.D.	N.A.	N.D.	N.A.	N.D.	N.A.
UDP	56.0	6.0	45.0	16.0	58.0	26.0	46.0	24.0	49.0	12.0	43.0	32.0
UMP	59.0	14.0	90.0	16.0	131.0	57.0	88.0	21.0	110.0	20.0	98.0	32.0
UTP	34.0	20.0	16.0	11.0	44.0	44.0	22.0	28.0	20.0	14.0	21.0	4.8
Uracil	N.D.	N.A.	N.D.	N.A.	N.D.	N.A.	N.D.	N.A.	N.D.	N.A.	N.D.	N.A.
Uridine	23.0	1.8	26.0	4.4	36.0	5.2	39.0	3.4	47.0	0.7	38.0	16.0
Val	294.0	13.0	336.0	87.0	381.0	58.0	350.0	103.0	377.0	61.0	394.0	77.0
cAMP	N.D.	N.A.	N.D.	N.A.	N.D.	N.A.	N.D.	N.A.	N.D.	N.A.	N.D.	N.A.
cGMP	N.D.	N.A.	N.D.	N.A.	N.D.	N.A.	N.D.	N.A.	N.D.	N.A.	N.D.	N.A.
cis-Aconitic acid	N.D.	N.A.	N.D.	N.A.	N.D.	N.A.	N.D.	N.A.	N.D.	N.A.	N.D.	N.A.
dATP	N.D.	N.A.	N.D.	N.A.	N.D.	N.A.	N.D.	N.A.	N.D.	N.A.	N.D.	N.A.
dCTP	N.D.	N.A.	N.D.	N.A.	N.D.	N.A.	N.D.	N.A.	N.D.	N.A.	N.D.	N.A.
dTDP	N.D.	N.A.	N.D.	N.A.	N.D.	N.A.	N.D.	N.A.	1.5	N.A.	N.D.	N.A.
dTMP	0.7	0.2	0.8	0.4	1.2	0.2	1.1	0.5	1.9	0.9	1.4	0.5
dTTP	N.D.	N.A.	N.D.	N.A.	N.D.	N.A.	N.D.	N.A.	N.D.	N.A.	N.D.	N.A.
β-Ala	443.0	120.0	385.0	143.0	1,282.0	751.0	779.0	42.0	796.0	609.0	902.0	457.0

N.D.: Not detected
N.A.: Not available

Appendix S3 Comparative statistical analysis of major metabolites using Welch's test.

Unfed (largest) vs. Unfed (smallest)

Compound name	*P*-value[1]	Ratio[2]
Phe	0.0012**	0.6
Ala	0.0053**	0.5
Pro	0.0054**	0.3
Gly	0.0073**	0.4
Trp	0.0075**	0.6
Sedoheptulose 7-phosphate	0.0133*	0.5
Glu	0.0142*	0.8
S-Adenosylmethionine	0.0240*	0.8
GABA	0.0242*	0.7
Betaine	0.0298*	1.3
Putrescine	0.0303*	0.5
GMP	0.0310*	0.6
Ribose 5-phosphate	0.0358*	0.6
6-Phosphogluconic acid	0.0382*	0.6
Uridine	0.0397*	0.6
dTMP	0.0472*	0.6
Dihydroxyacetone phosphate	0.0482*	0.4
Glucose 1-phosphate	0.0484*	0.5

Unfed (largest) vs. *C. neogracile* (smallest)

Compound name	*P*-value[1]	Ratio[2]
Uridine	0.0056**	0.6
Lactic acid	0.0131*	0.6
Citric acid	0.0150*	0.6
Spermidine	0.0158*	0.7
Dihydroxyacetone phosphate	0.0200*	0.5
Glu	0.0225*	0.8
β-Ala	0.0292*	0.6
Arg	0.0378*	1.3
Choline	0.0432*	0.7

Appendix S3 Comparative statistical analysis of major metabolites using Welch's test. (Continued 1)

Unfed (largest) vs. *C. neogracile* + AHs (largest)

Compound name	*P*-value[1]	Ratio[2]
Uridine	0.0005***	0.5
Guanine	0.0014**	0.4
Gly	0.0018**	0.3
Pro	0.0026**	0.4
Ser	0.0049**	0.6
Gln	0.0058**	0.2
Dihydroxyacetone phosphate	0.0114*	0.4
Ala	0.0133*	0.6
Gluconic acid	0.0234*	0.6
GMP	0.0244*	0.5
Citrulline	0.0285*	0.6
UMP	0.0293*	0.5
Putrescine	0.0312*	0.6
Sedoheptulose 7-phosphate	0.0381*	0.5
Spermidine	0.0464*	0.7

Unfed (largest) vs. *C. neogracile* + AHs (smallest)

Compound name	*P*-value[1]	Ratio[2]
Dihydroxyacetone phosphate	0.0074**	0.4
His	0.0098**	0.5
Succinic acid	0.0192*	0.5
Fructose 1,6-diphosphate	0.0195*	0.2
GABA	0.0246*	0.5
CMP	0.0275*	0.7
GMP	0.0334*	0.6
Gly	0.0446*	0.5
Ala	0.0456*	0.7
Putrescine	0.0471*	0.6

Appendix S3 Comparative statistical analysis of major metabolites using Welch's test. (Continued 2)

Unfed (smallest) vs. *C. neogracile* (largest)

Compound name	*P*-value[1]	Ratio[2]
Phe	0.0008***	0.7
Fructose 6-phosphate	0.0023**	0.4
Pro	0.0032**	0.3
Glucose 1-phosphate	0.0038**	0.4
Gly	0.0041**	0.3
Betaine	0.0060**	1.4
Trp	0.0063**	0.7
Ala	0.0068**	0.5
Glucose 6-phosphate	0.0115*	0.5
Sedoheptulose 7-phosphate	0.0182*	0.4
6-Phosphogluconic acid	0.0253*	0.6
Fumaric acid	0.0270*	0.5
Ribulose 5-phosphate	0.0398*	0.6
Asp	0.0436*	0.7
Dihydroxyacetone phosphate	0.0472*	0.4

Unfed (smallest) vs. *C. neogracile* (smallest)

Compound name	*P*-value[1]	Ratio[2]
Uridine	0.0179*	0.7
Spermidine	0.0241*	0.6
Malic acid	0.0315*	0.6
Dihydroxyacetone phosphate	0.0320*	0.5
β-Ala	0.0327*	0.5
ADP	0.0465*	0.7

Appendix S3 Comparative statistical analysis of major metabolites using Welch's test. (Continued 3)

Unfed (smallest) vs. *C. neogracile* + AHs (largest)

Compound name	*P*-value[1]	Ratio[2]
Gly	0.0029**	0.003
Guanine	0.0033**	0.003
Pro	0.0080**	0.008
Gln	0.0086**	0.009
Uridine	0.0134*	0.013
Betaine	0.0136*	0.014
Glutathione (GSSG)_divalent	0.0138*	0.014
Fumaric acid	0.0150*	0.015
Gluconic acid	0.0153*	0.015
Ala	0.0171*	0.017
Fructose 6-phosphate	0.0185*	0.019
Dihydroxyacetone phosphate	0.0205*	0.021
Ser	0.0253*	0.025
Malic acid	0.0319*	0.032
Citrulline	0.0348*	0.035
Sedoheptulose 7-phosphate	0.0413*	0.041
Spermidine	0.0427*	0.043
Glucose 6-phosphate	0.0428*	0.043
CDP	0.0484*	0.048

Unfed (smallest) vs. *C. neogracile* + AHs (smallest)

Compound name	*P*-value[1]	Ratio[2]
His	0.0093**	0.5
Dihydroxyacetone phosphate	0.0156*	0.4
Fructose 1,6-diphosphate	0.0224*	0.2
Gly	0.0241*	0.3
Succinic acid	0.0337*	0.6

C. neogracile (largest) vs. *C. neogracile* (smallest)

Compound name	*P*-value[1]	Ratio[2]
Lactic acid	0.0117*	0.6
Sedoheptulose 7-phosphate	0.0125*	1.4
Fructose 6-phosphate	0.0137*	1.6
Glucose 6-phosphate	0.0249*	1.6
Glucose 1-phosphate	0.0272*	1.7

Appendix S3 Comparative statistical analysis of major metabolites using Welch's test. (Continued 4)

C. neogracile (largest) vs. *C. neogracile* + AHs (largest)

Compound name	*P*-value[1]	Ratio[2]
Pyruvic acid	0.0041**	1.3
Phosphoenolpyruvic acid	0.0294*	0.7

C. neogracile (largest) vs. *C. neogracile* + AHs (smallest)

Compound name	*P*-value[1]	Ratio[2]
Phosphoenolpyruvic acid	0.0082**	0.6
3-Phosphoglyceric acid	0.0186*	0.7
Succinic acid	0.0400*	0.6
Ala	0.0420*	1.3
PRPP	0.0427*	1.6
2-Phosphoglyceric acid	0.0474*	0.8

C. neogracile (smallest) vs. *C. neogracile* + AHs (largest)

Compound name	*P*-value[1]	Ratio[2]
Ser	0.0012**	0.6
N,N-Dimethylglycine	0.0017**	4.0
Arg	0.0046**	0.7
Lactic acid	0.0269*	1.5
IMP	0.0394*	0.3
Phosphoenolpyruvic acid	0.0439*	0.7
Guanine	0.0486*	0.6

C. neogracile (smallest) vs. *C. neogracile* + AHs (smallest)

Compound name	*P*-value[1]	Ratio[2]
Phosphoenolpyruvic acid	0.0154*	0.6
3-Phosphoglyceric acid	0.0399*	0.7
Arg	0.0446*	0.8

C. neogracile + AHs (largest) vs. *C. neogracile* + AHs (smallest)

Compound name	*P*-value[1]	Ratio[2]
2-Phosphoglyceric acid	0.0029**	0.8
3-Phosphoglyceric acid	0.0056**	0.9
Ser	0.0347*	1.6

[1] $*P < 0.05$, $**P < 0.01$, and $***P < 0.001$.
[2] For calculation of the ratios, the value for the first group in the comparison was divided by the value for the second.
